# State-Interaction Approach for *g*‑Matrix Calculations in TDDFT: Ground-Excited State Couplings and beyond First-Order Spin–Orbit Effects

**DOI:** 10.1021/acs.jctc.5c00514

**Published:** 2025-06-18

**Authors:** Antonio Cebreiro-Gallardo, David Casanova

**Affiliations:** † 226245 Donostia International Physics Center (DIPC), 20018 Donostia, Euskadi, Spain; ‡ Polimero eta Material Aurreratuak: Fisika, Kimika eta Teknologia Saila, Kimika Fakultatea, Euskal Herriko Unibertsitatea (UPV/EHU), PK 1072, 20080 Donostia, Euskadi, Spain; § IKERBASQUE, Basque Foundation for Science, 48009 Bilbao, Euskadi, Spain

## Abstract

We introduce a state-interaction approach for computing *g*-matrices within time-dependent density functional theory (TDDFT) and the Tamm–Dancoff approximation (TDA), applied here for the first time. This method provides a detailed understanding of *g*-shifts by explicitly accounting for spin–orbit couplings (SOC) and excitation energies, enabling the analysis of different SOC orders and their contributions. To evaluate its accuracy and reliability, we compare state-interaction TDDFT and TDA with the widely used one-component coupled-perturbed Kohn–Sham approach. Applications to a diverse set of systems, including light and heavy atom molecules as well as transition-metal complexes, demonstrate that both methods yield comparable results in the absence of heavy elements, while the state-interaction approach offers improved insights into SOC effects and their impact on *g*-shifts.

## Graphical abstract

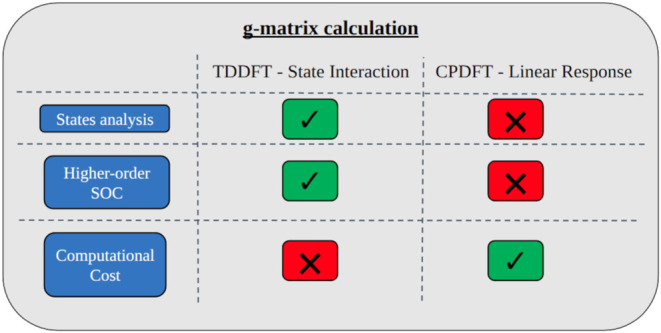

## Introduction

Electron paramagnetic resonance (EPR) spectroscopy is a powerful technique for studying paramagnetic systems containing unpaired electrons, offering insights into their electronic and geometrical structures and their local environments.^1^ The technique involves the interaction between the sample and an external magnetic field, which causes the splitting of the energy levels due to the Zeeman effect. When the energy of the photons probed matches the energy difference between the spin states, absorption occurs and the EPR signal is detected.

The EPR spectrum is typically analyzed using a spin Hamiltonian, an effective Hamiltonian that describes energy level splittings through a set of empirical parameters.^2^ A key parameter in this framework is the *g*-matrix (often referred to as the *g*-tensor, although it is not a true tensor),^3^ which quantifies the interaction between the total electronic magnetic moment of the system and an external magnetic field. As the *g*-matrix effectively summarizes electronic transitions in EPR spectra, its accurate computation and characterization are crucial for understanding the electronic structure of diverse systems, including organic radicals, open-shell transition-metal ions, and molecular magnets.^4–7^

Over the past 70 years, various methodologies have been developed to determine the *g*-matrix.^8^ For a long time, semiempirical methods and Hückel-type approximations were the primary approaches.^9^ However, these methods rely on parametrizations that are only valid for specific systems and often involve coarse approximations. Since the 1990s, significant advances have been made with the development and application of both wave function theory (WFT) and density functional theory (DFT) methods for computing *g*-parameters, leading to more accurate and broadly applicable predictions.

Early first-principles calculations of the *g*-matrix were performed at the Hartree–Fock (HF) level,^10,11^ though they relied on an incomplete spin Hamiltonian. A significant advancement came from Lushington and Grein, who introduced an *ab initio* treatment incorporating a spin Hamiltonian up to second order at the restricted open-shell Hartree–Fock (ROHF) level using Rayleigh–Schrödinger perturbation theory.^12^ They also demonstrated that including both static and dynamic electron correlation, through multireference configuration interaction (MR-CI) combined with sum-overstates (SOS) expansions, greatly improved the accuracy of *g*-matrix calculations, particularly for small molecules.^13^ However, a major limitation of this approach is the truncation in the number of excited states considered in the SOS expansion. To address this, Vahtras and co-workers developed an alternative methodology based on response theory, which implicitly accounts for all electronic states, overcoming the truncation issue.^14^ Further developments in the calculation of *g*-matrices introduced various methodological advancements across different electronic structure approaches. Jayatilaka proposed a HF procedure based on the Hellmann–Feynman theorem, avoiding the need for a perturbative treatment.^15^ Pierloot and co-workers extended multireference methods by incorporating second-order perturbation theory (MR-PT2).^16^ Within the MR-CI framework, Brownridge and colleagues developed a SOS approach,^17^ while Neese introduced both an SOS-based method and an analytical derivative-based MR-CI approach.^18,19^ Bolvin^20^ placed the nonperturbative formulation of Gerloch and McMeeking’s equations^21^ within the broader context of modern *g*-matrix calculations. In the coupled-cluster (CC) framework, Gauss et al. developed a methodology to compute *g*-matrices with high accuracy.^22^ Tatchen et al. proposed an approach for computing *g*-matrices using multireference spin–orbit configuration interaction (MR-SOCI), which can be extended to systems with higher spin multiplicities.^23^ More recently, the calculation of *g*-parameters with a state-interaction approach has been extended to equation-of-motion CC (EOM-CC) methods and restricted active space CI (RASCI) wave functions.^24,25^ Lan et al. provided an insightful comparison between different perturbation theory treatments within the complete-active space self-consistent field (CASSCF) method, contrasting first-order quasi-degenerate perturbation theory (QDPT) with second-order linear-response theory implemented via coupled–perturbed CASSCF (CP-CASSCF). Higher-level correlation effects have been incorporated in accurate *g*-matrix calculations, particularly through multiconfigurational perturbation theory.^26,27^

In parallel, significant efforts have been dedicated to developing methodologies for calculating the *g*-matrix within the density functional theory (DFT) framework. The success of DFT in chemical applications stems from its Kohn–Sham (KS) formulation, which offers a favorable balance between accuracy and computational cost, making it an attractive alternative to WFT-based approaches,^28^ particularly for larger molecular systems and in the calculation of molecular properties, such as the *g*-parameters.^8^ However, a well-known and challenging aspect of KS-DFT is its dependence on the choice of the exchange–correlation (XC) functional, which can influence the accuracy of *g*-matrix calculations.^29^ In particular, DFT tends to underestimate *g*-shifts in transition-metal ion complexes,^30–34^ primarily due to an overestimation of covalency in chemical bonds. This effect originates from the well-known delocalization error in DFT, which can lead to inaccurate descriptions of electronic structures.^35^ Additionally, standard DFT faces inherent challenges in describing systems with significant multireference character and in accurately treating spin–orbit two-electron interactions, both of which are crucial for precise *g*-matrix calculations.^8,26^ These limitations highlight the need for improved functionals and hybrid approaches that incorporate WFT correlation effects to enhance the accuracy of DFT-based *g*-matrix predictions.

Depending on how SOC is incorporated, methodologies for computing *g*-parameters can be categorized into one-, two-, or four-component approaches. In one-component DFT, SOC is treated as a perturbation and the *g*-shift is computed as a second-order property. This can be formulated either through linear-response theory or as a SOS expansion,^36^ both of which account only for linear SOC effects.^37^ As a result, these approaches are well-suited for systems in which SOC is relatively weak, such as high-spin molecules composed of light elements. However, they are generally inadequate for systems containing heavy elements, where second- and higher-order SOC effects become significant.

The first study dedicated to the computation of the *g*-matrix using one-component DFT methods was conducted by Schreckenbach and Ziegler in 1997,^38^ where they also pioneered the use of gauge-including atomic orbitals (GIAO). Their implementation incorporated all of the relevant perturbation operators except for spin-other-orbit interactions. Building on this work, Patchkovskii and Ziegler applied the method to a series of axially symmetric pentacoordinated transition-metal complexes,^39^ later generalizing it to systems with higher multiplicities beyond doublet Kramers-type radicals.^40^ Subsequent advancements included the incorporation of hybrid functionals into *g*-matrix calculations, introduced independently by Malkin et al.^41^ and Neese.^30^ In this context, Neese implemented coupled-perturbed HF and KS methods to obtain the *g*-values. Within this framework, the *g*-parameters are evaluated as a second derivative property with respect to both the external magnetic field and the electronic spin. This approach, combined with different families of functionals, has demonstrated a high accuracy for small radicals and transition-metal complexes. A further refinement in one-component methodologies came from Malkina et al., who were the first to apply the mean-field approximation to the full Breit–Pauli (BP) spin–orbit Hamiltonian in *g*-matrix calculations.^42^ The effects of various approximations to the full BP SOC operator were later systematically investigated by Neese.^43^ Advancing beyond these approximations, Van Yperen-De Deyne et al. proposed an improved description of spin–other-orbit interactions, which is particularly important in high-spin systems, along with a protocol to enhance the treatment of exchange spin–orbit interactions.^44^

In two-component DFT methods, *g*-shifts are evaluated as first-order properties. Spin–orbit coupling (SOC) is incorporated variationally within the wave function, implicitly accounting for higher-order SOC effects, while the external magnetic field is treated as a perturbation. A significant advancement in this area came with the variational DFT-GIAO implementation using the zero-order regular approximation (ZORA) for relativistic effects, which includes SOC at a variational level. This approach was first reported by Van Lenthe^45^ and later extended by Belanzoni.^46^ A comparative study between ZORA calculations performed with the ADF and NWChem codes, testing various density functionals, was carried out by Aquino et al.^47^ Malkin et al. introduced a relativistic approach that incorporates spin polarization and higher-order SOC effects in *g*-tensor calculations, demonstrating its ability to reproduce phenomena such as negative *g*-values.^48^ Recognizing the significance of higher-order effects in *g*-shift computations, Komorovský and collaborators developed an efficient relativistic two-component DFT method (DKS2-RI), which enables the calculation of *g*-parameters for heavy elements while avoiding picture-change effects.^49^ Further contributions were made by Cherry and co-workers, who described an implementation of relativistic unrestricted two- and four-component approaches and analyzed the challenges associated with using an unrestricted framework.^50^ Additionally, Neyman et al. presented a scheme for computing electronic *g*-tensors of doublet states within a DFT framework, utilizing two-component eigenfunctions of the KS equation, where SOC is treated self-consistently within the Douglas–Kroll formalism.^51^ In the late 2010s,^52^ exact two-component (X2C) method was applied to the calculation of magnetic properties, including *g*-tensors, demonstrating high accuracy and further methodological advancements and refinements have solidified its role as a reliable tool for incorporating scalar and spin–orbit relativistic effects in quantum chemical calculations.^53^

Four-component relativistic methods represent the most rigorous framework for *g*-tensor calculations, with key milestones including the first implementation within pure DFT,^54^ the extension to hybrid DFT functionals,^55^ and the incorporation of restricted magnetic balance and gauge-including atomic orbitals (GIAOs) to ensure gauge-origin invariance and improved accuracy.^56^

Alternatively, second- and higher-order SOC effects can be incorporated within one-component methods using quasi-degenerate perturbation theory (QDPT), also known as the state-interaction approach. This strategy is particularly valuable as it enables the characterization of computed *g*-shifts in terms of interstate interactions mediated by SOC. While this approach has been extensively explored with various wave function-based methods,^20,23–25,57^ it has not yet been applied to nonrelativistic states obtained from DFT-based methods. In this work, we introduce and implement a state-interaction approach within the DFT framework, where SOC effects are incorporated through interactions between nonrelativistic KS-DFT and time-dependent DFT (TDDFT) roots. This formulation not only allows for a detailed characterization of computed *g*-shifts but also systematically includes second- and higher-order SOC contributions. Compared to state-interaction approaches in wave function theory, the proposed methodology retains the simplicity and computational efficiency of DFT in its linear-response formulation, making it particularly well-suited for large molecular systems. To assess its accuracy and reliability, our state-interaction TDDFT methodology will be benchmarked against experimental measurements and the one-component coupled-perturbed KS (CPKS) technique developed by Neese.^30^

The review is organized as follows. The Theoretical Background section provides an overview of the theoretical background, introducing the foundations of the state-interaction TDDFT method and summarizing the key features of the CPKS approach for completeness. The Computational Details section details the computational methodologies employed in this study. In the Results and Discussion section, we assess the performance of state-interaction TDDFT by comparing its results against CPKS calculations and experimental data across different molecular systems, including small light molecules, small heavy molecules, and transition-metal complexes. Finally, the Conclusions section presents the conclusions.

## Theoretical Background

### State-Interaction TDDFT Approach

In state-interaction approaches, the *g*-matrix parameters are obtained through the mapping of the Zeeman Hamiltonian (eq 1) with an effective spin Hamiltonian (eq 2)­

1 $$H_{\mathrm{Zeeman}}=\beta_{e}B^{t}\left(L+g_{e}S\right)$$

2 $$H_{\mathrm{spin}}=\beta_{e}B^{t}g\mathrm{S̃}$$

where β_e_ is the Bohr magneton, *g*_e_ is the free-electron isotropic *g*-factor, **B** is the external magnetic field vector, and **L** and **S** are the electronic orbital and spin vector operators, respectively, **g** is a matrix containing the strength and anisotropy of the Zeeman interaction, and **S̃** is the effective spin (pseudospin) vector.

Spin–orbit relativistic effects are incorporated as couplings between the (spin-pure) eigenstates of the nonrelativistic Hamiltonian, specifically the KS configuration and TDDFT states. To obtain the relativistic states, we employ a QDPT approach, following a “perturb-then-diagonalize” scheme. This involves constructing and diagonalizing a SOC-dressed effective Hamiltonian

3 $$H_{\mathrm{IJ}}^{\mathrm{eff}}=E_{I}\delta_{\mathrm{IJ}}+H_{\mathrm{IJ}}^{\mathrm{SO}}$$

where *I* and *J* denote nonrelativistic KS and TDDFT states with {*E*_*I*_} eigenenergies, and *H*_*IJ*_^SO^ = ⟨*I* |*H* ^SO^|*J*⟩ is the SOC between *I* and *J* states. Diagonal values in eq 3 are obtained as excitation energies from (linear-response) TDDFT^58–62^ with and without the Tamm–Dancoff approximation (TDA),^63^ while off-diagonal elements correspond to SOCs computed between TDA states, as implementation of full mean-field SOCs between TDDFT states based on the Wigner–Eckart theorem is not currently available.

SOCs between KS and TDA nonrelativistic states are computed with the BP Hamiltonian

4 $$H_{\mathrm{BP}}^{\mathrm{SO}}=\frac{1}{2c^{2}}\left[\sum_{i}h^{\mathrm{SO}}\left(i\right)\cdot s\left(i\right)+\sum_{i\neq j}h^{\mathrm{SOO}}\left(i,j\right)\cdot \left(s\left(i\right)+2s\left(j\right)\right)\right]$$

5 $$h^{SO}\left(i\right)=\sum_{I}\frac{Z_{A}}{r_{iA}^{3}}\left(r_{iA}\times p_{i}\right)$$

6 $$h^{\mathrm{SOO}}\left(i,j\right)=-\frac{1}{r_{\mathrm{ij}}^{3}}\left(r_{\mathrm{ij}}\times p_{i}\right)$$

where *c* is the speed of light, **s**(*i*) is the spin operator of the *i*th electron, *Z*_*A*_ is the nuclear charge of the *A*th nucleus, **r**_*iA*_ is the distance between electron *i* and nucleus *A*, **p**_*i*_ is the momentum operator, and **h** ^SO^(*i*) and **h** ^SOO^ are one- and two-electron spin–orbit operators defined in eqs 5 and 6.

The two-electron terms in the BP Hamiltonian are often approximated using the spin–orbit mean-field (SOMF) approach.^64^ In this work, we adopt the mean-field implementation within the TDDFT framework recently introduced by Kotaru and collaborators,^65^ where the use of the Wigner–Eckart theorem enables to express the SOC between electronic states as

7 $$\left\langle \mathrm{ISM}\right|H_{\mathrm{BP}}^{\mathrm{SOMF}}\left|I^{\prime }S^{\prime }M^{\prime }\right\rangle =\frac{1}{2}\sum_{\mathrm{pq}}\left[\langle S^{\prime }M^{\prime };1-1\left|\mathrm{SM}\right\rangle h_{L_{+},\mathrm{pq}}^{\mathrm{SOMF}}+\langle S^{\prime }M^{\prime };10\left|\mathrm{SM}\right\rangle \sqrt{2}h_{z,\mathrm{pq}}^{\mathrm{SOMF}}-\langle S^{\prime }M^{\prime };11\left|\mathrm{SM}\right\rangle h_{L_{-},\mathrm{pq}}^{\mathrm{SOMF}}\right]u_{\mathrm{pq}}$$

where *I*, *S*, *M* indices indicate *I*th electronic state with spin *S* and spin projection *M*, ⟨*S*′*M*′;1*M*″|*SM*⟩ is a Clebsh–Gordan coefficient, *h*_*z*,*pq*_^SOMF^, *h*_*L*_+_,*pq*_^SOMF^ and *h*_*L*_–_,*pq*_^SOMF^ contain the sum of one-electron and mean-field two-electron interaction terms,^66^ and *u* is a spinless triplet transition density matrix,^67^ which can be constructed through the spin-conserving αα and ββ parts of the one-particle transition density matrix (γ) between the state with the same spin projection (*M*)­

8 $$u_{\mathrm{pq}}=\frac{\left(\gamma_{\mathrm{pq}}-\gamma_{\mathrm{p̅}\mathrm{q̅}}\right)}{\sqrt{2}\left\langle S^{\prime }M;10\right|\mathrm{SM}\rangle }$$

9 $$\gamma_{\mathrm{pq}}=\left\langle \mathrm{ISM}\right|{â}_{p}^{†}{â}_{q}\left|I^{\prime }S^{\prime }M\right\rangle$$

It is important to notice that eq 8 assumes spin-pure states, which might not be the case of spin-unrestricted KS high-spin ground states and linear-response TDA roots. Therefore, our approach includes spin polarization effects through the use of unrestricted orbitals but disregards the spin contamination of electronic states. We assume that this will be a reasonable approach when considering states with small or moderate spin contamination. To assess its validity, computed ⟨*Ŝ* ^2^⟩ values for the KS ground state and the most relevant TDA excited states of all studied molecules are provided in the Supporting Information (Table S2).

A key advantage of eq 7, in combination with eqs 8 and 9, is that it eliminates the need to explicitly compute all spin sublevels for each electronic state in the state-interaction expression. Instead, it suffices to calculate only a single spin projection (*M*′ = *M*). Specifically, since electronic states can be computed using the same spin projection for all states, as done in eqs 8 and 9, they can be obtained from a single TDA calculation.

Finally, the *g*-matrix is determined by equating the Zeeman Hamiltonian (eq 1) with the spin Hamiltonian (eq 2). This requires expressing the angular momentum (**L**) and spin (**S**) operators in the basis of the target multiplet spin–orbit coupled states, specifically, the one mainly constituted by the ground state triplet of the nonrelativistic Hamiltonian. The extraction of *g*-parameters is performed using the projection technique introduced by Tatchen and co-workers.^23^

### Coupled-Perturbed Kohn–Sham

For completeness, we summarize the key expressions of the CPKS method for *g*-matrix calculations,^30^ as this approach will be systematically compared with our state-interaction TDDFT/TDA results. In CPKS theory, the *g*-shift elements, defined as Δ*g*_rs_ = *g*_rs_ – *g*_e_δ_rs_, are decomposed into three distinct contributions^8,68^

10 $$\Delta g_{\mathrm{rs}}=\Delta g_{\mathrm{rs}}^{\mathrm{RMC}}\delta_{\mathrm{rs}}+\Delta g_{\mathrm{rs}}^{\mathrm{GC}}+\Delta g_{\mathrm{rs}}^{\mathrm{OZ}/\mathrm{SOC}}$$

where Δ*g* ^RMC^ represents the relativistic mass correction, Δ*g* ^GC^ corresponds to the gauge correction, also called the diamagnetic correction, and Δ*g* ^OZ/SOC^ accounts for the spin–orbit and orbital Zeeman contributions. The dominant contribution to Δ*g* comes from the OZ/SOC term, Δ*g* ^OZ/SOC^, a second-order correction arising from the interplay between SOC and the orbital Zeeman interaction. Notably, this term is equivalent to the SOC effects incorporated through the state-interaction methodology described above, effectively capturing how an external magnetic field influences the orbital motion of electrons, further modulated by SOC.

The CPKS approach relies on linear-response theory, treating both SOC and the Zeeman interaction as perturbations to a field-free Hamiltonian.^30^ Specifically, the contribution of the OZ/SOC to the *g*-matrix is obtained as the second derivative of the energy with respect to the external magnetic field and the electronic spin magnetic moment (μ_s_), employing a mean-field approximation to the Breit–Pauli Hamiltonian

11 $$\Delta g_{\mathrm{rs}}^{\mathrm{OZ}/\mathrm{SOC}}=\frac{1}{\beta_{e}S}\frac{\partial^{2}E}{\partial B_{r}\partial \mu_{s}}$$

## Computational Details

Ground-state optimized geometries of small light molecules (H_2_O^+^, NO_2_, and CO_2_^–^) were taken from ref 30. Optimized coordinates of NBr, PdH, CdH, HgH, RhH_2_ and IrH_2_ molecules are taken from ref 57, and bond distance of NI was taken from ref 40. Molecular structures of transition-metal complexes [CuCl_4_]^2−^ and [Cu­(NH_3_)_4_]^2+^ were taken from ref 69; TiF_3_ and [Ni­(mnt)_2_]^−^ from ref 30; and the [MOX_4_]^*n*−^ (M = V, Cr, Mo; X = F, Cl, Br) series from ref 26.

Hybrid and generalized gradient approximation (GGA) functionals yield comparable predictions for light radicals.^30^ However, in transition-metal complexes, the accuracy of a given functional can vary significantly across different classes of compounds.^70^ Hybrid functionals generally outperform pure local density approximation (LDA) and GGA functionals,^41^ primarily due to their improved treatment of metal–ligand covalency. In this study, unless stated otherwise, we systematically employ the B3LYP hybrid GGA functional (with the VWN3 parametrization for the local correlation component), as it has been shown to provide results that closely align with experimental data.^30^ In all cases, an energy convergence threshold of 1 × 10^–8^ Ha was used for the SCF procedure, while an orbital-pair overlap threshold of 1 × 10^–12^ was enforced for the evaluation of two-electron integrals. Additionally, the resolution of identity (RI) approximation, which is the default option in ORCA, was explicitly disabled in all calculations.

The relatively low computational cost of TDDFT (and TDA) allows for the inclusion of a large number of electronic states in the spin–orbit-dressed Hamiltonian (eq 3). Unless otherwise specified, the state-interaction protocol was evaluated using 100 excited electronic states. The convergence of the computed *g*-shifts was assessed by analyzing individual state contributions and the dependence of *g*-shifts on the size of the effective Hamiltonian. Since our state-interaction approach relies on the calculation of electronic excited states, achieving accurate results requires basis sets flexible enough to describe electronic transitions properly. Previous studies suggest that reliable molecular *g*-matrix predictions require at least triple-ζ basis sets,^38,44,69,71,72^ while the inclusion of diffuse functions has little to no impact on accuracy.^17^ For small molecules and transition-metal complexes, we use the def2-TZVP basis set,^73^ combined with the effective core potential (def2-ECP) for elements from Rb to Rn. In contrast, heavy-element-containing molecules require basis sets that account for relativistic effects.^25,57^ Thus, we employ the ANO-RCC-TZVP basis set^74,75^ for the small heavy-element molecules NBr, NI, PdH, CdH, HgH, RhH_2_, and IrH_2_.

The gauge dependence of the *g*-matrix, arising from the origin dependence of the angular momentum operator in finite basis sets, has been a longstanding concern in theoretical studies.^76^ The most accurate approach to achieve gauge invariance is the use of gauge-including atomic orbitals (GIAOs).^38^ Selecting the electronic charge centroid as a common gauge origin,^12,20,77^ as proposed by Luzanov et al.,^78^ or the center of nuclear charges^79^ has also yielded reliable results. In light atoms, the choice of gauge origin has little effect on the *g*-matrix when large basis sets with polarization functions are used.^80^ The impact is also minimal when *g*-shifts are significant, on the order of parts per thousand.^42^ However, in larger molecules, a common gauge origin can lead to large errors even with large basis sets.^81^ Table S6 in the Supporting Information includes the *g*-shifts computed with CPKS using the center of nuclear charge as common gauge origin or using GIAOs, showing there are no relevant differences in the transition-metal complexes. For this reason, the center of nuclear charge has been chosen as the common gauge origin in all of the studied molecules.

SOC effects have been computed using the BP Hamiltonian within the mean-field approximation for two-electron integrals, a method that has demonstrated excellent performance for molecules containing light to moderately heavy elements.^24,25,43,44^ However, it is important to note that the accuracy of the BP Hamiltonian diminishes for heavier elements and is generally inadequate for elements with large atomic numbers.^38,82,83^ SOCs computations were performed using the frozen core approximation.

TDDFT and TDA electronic structure calculations have been carried out using a developer’s version of the Q-Chem program.^84^ CPKS calculations have been performed with ORCA 6.0.^43,85–87^ Evaluation of the *g*-matrix parameters was carried out with in-house codes integrated within the PyQChem interface.^88^

## Results and Discussion

To evaluate the state-interaction protocol within the TDDFT/TDA framework, we compute and analyze the *g*-matrix components for a diverse set of molecules. This includes three light-atom triatomic species (H_2_O^+^, NO_2_, and CO_2_^–^), several diatomic and triatomic molecules containing heavy elements (NBr, NI, PdH, CdH, HgH, RhH_2_, and IrH_2_), and a series of transition-metal complexes.

### Light Atom Molecules

Table 1 presents the *g*-shifts for the spin-doublet ground states of triatomic molecules H_2_O^+^, NO_2_, and CO_2_^–^. Overall, the TDA and TDDFT results closely match their CPKS counterparts and show good agreement with experimental values. TDDFT exhibits slightly better accuracy than CPKS for the charged species but performs less well for NO_2_. The largest deviations from experiment occur for Δ*g*_*yy*_ of H_2_O^+^, in line with previous computational studies.^13,17,89^

**Table 1:** Calculated Δ*g* Values (in ppt) for H_2_O^+^, NO_2_, and CO_2_^–^ Molecules Obtained with CPKS, TDA, and Full TDDFT Methods with the def2-TZVP Basis Set

molecule		CPKS	TDA	TDDFT	exp.^a^
H_2_O^+^	Δ*g*_*xx*_	–0.2	–0.4	–0.4	0.2
	Δ*g*_*yy*_	12.6	13.7	14.3	18.8
	Δ*g*_*zz*_	4.2	4.2	4.3	4.8
NO_2_	Δ*g*_*xx*_	3.6	2.4	2.5	3.9
	Δ*g*_*yy*_	–10.9	–10.4	–10.7	–11.3
	Δ*g*_*zz*_	–0.6	–1.4	–1.4	–0.3
CO_2_^–^	Δ*g*_*xx*_	1.4	1.0	1.0	0.7
	Δ*g*_*yy*_	–5.1	–4.9	–5.0	–4.8
	Δ*g*_*zz*_	–0.7	–0.8	–0.8	–0.5

a Experimental results from ref 30.

The state-interaction procedure with TDDFT allows for a detailed description and rationalization of the components of the *g*-matrix in terms of the interactions between the ground and excited states. All three molecules under study belong to the *C*_2*v*_ symmetry point group, and each component of Δ*g* primarily originates from a limited number of excited states, as shown in Figure S9 in the Supporting Information. In H_2_O^+^, the *g*-shifts arise from the SOC of the X^2^ *B*_2_ ground state with several excited states: 1^2^ *A*_1_ for Δ*g*_*yy*_, 1^2^ *B*_1_ for Δ*g*_*zz*_, and multiple low-lying ^2^ *A*_2_ states contributing to the small Δ*g*_*xx*_, consistent with the results of Vancoillie et al.^26^ In NO_2_, *g*-shifts along the *x*-, *y*-, and *z*-directions are induced by the interaction of the X^1^ *A*_1_ ground state with ^2^ *B*_1_, ^2^ *B*_2_, and ^2^ *A*_2_ states, respectively, as previously noted by others.^17,24^ As Lushington and Grein observed,^13^ Δ*g*_*yy*_ primarily originates from a single excited state contribution, while Δ*g*_*xx*_ and Δ*g*_*zz*_ emerge from several excited states (see Figure S9). The *g*-shifts in CO_2_^–^ result from the interaction between the X^2^A_1_ ground state and several low-lying ^2^B_1_ states for Δ*g*_*xx*_, in agreement with Brownridge’s MR-CI results.^17^ The largest contributions to Δ*g*_*yy*_ and Δ*g*_*zz*_ respectively come from the lowest ^2^B_2_ and ^2^A_2_ states.

We also employed these three molecules to investigate the influence of different DFT energy functionals on the calculation of molecular *g*-factors. To this end, we consider a total of 17 exchange–correlation functionals, encompassing a range of approximations: pure GGA functionals (e.g., BLYP), hybrid GGA functionals (e.g., B3LYP), long-range corrected hybrids (e.g., CAM-B3LYP), and functionals incorporating nonlocal correlation (e.g., ωB97X-V). Figure 1a presents the *g*-shifts of H_2_O^+^ computed with different functionals. Our results reveal no significant variation across the different exchange–correlation functionals with analogous trends observed for NO_2_ and CO_2_^–^ (Figures S10 and S11). These findings are consistent with the results of Verma and Autschbach, who reported similar *g*-factor predictions between HF and DFT for light main-group elements.^31^

**Figure 1:**
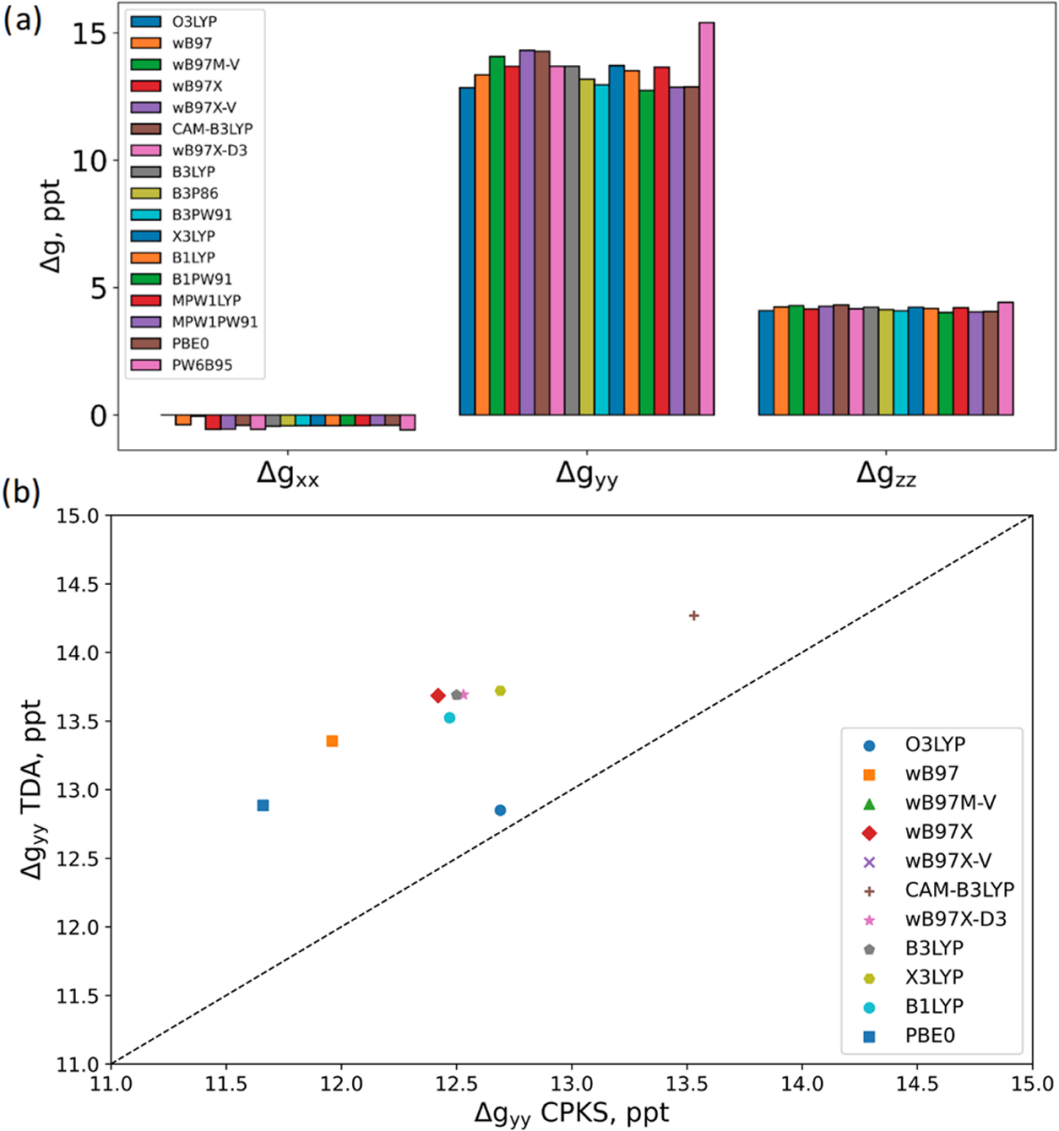
Δ*g* Components of H_2_O^+^ (a) computed at the TDA/def2-TZVP level with various exchange–correlation functionals and (b) a comparison between TDA and CPKS results with the same basis set and functionals.

Figure 1b compares the largest Δ*g* component, Δ*g*_*yy*_, in H_2_O^+^ as obtained from state-interaction TDA and CPKS. While TDA systematically predicts larger *g*-shifts, in general, both approaches exhibit similar trends concerning the choice of exchange–correlation functional. Furthermore, it is worth noting that within the CPKS framework, *g*-matrices incorporating the nonlocal component of the VV10 functional,^90^ as implemented in functionals such as ωB97X-V^91^ and ωB97M-V,^92^ can be evaluated either as an additive correction on a converged density or through a fully self-consistent procedure.^93,94^ While previous studies suggest that the choice between these two approaches has little impact on state energies,^94^ our results indicate that it significantly affects the computed Δ*g* values. In particular, the self-consistent approach yields results that are more consistent with those obtained by using other functionals (Table S3). For this reason, the results presented in Figure S3 for the ωB97X-V and ωB97M-V functionals include the fully self-consistent treatment of the nonlocal dispersion correction.

As indicated in eq 10, the Δ*g* values computed with CPKS include three contributions: the relativistic mass correction (RMC), the gauge correction (GC), and the OZ/SOC term. In contrast, our state-interaction TDDFT/TDA approach accounts only for the paramagnetic spin–orbit term (equivalent to OZ/SOC in CPKS), as this is known to be the dominant contribution.^39^ As shown in Figure 2 for NO_2_, RMC, and GC contributions are negligible compared to OZ/SOC, except in cases where the latter is very small—such as in Δ*g*_*zz*_—where the overall Δ*g* value is also minimal. This trend holds across all studied molecules (Table S4), justifying the direct comparison between the CPKS and TDDFT/TDA (SOC-only) Δ*g* results. Additionally, it is worth noting that two-electron contributions to OZ/SOC and GC partially cancel out their respective one-electron counterparts, which are consistently larger.

**Figure 2:**
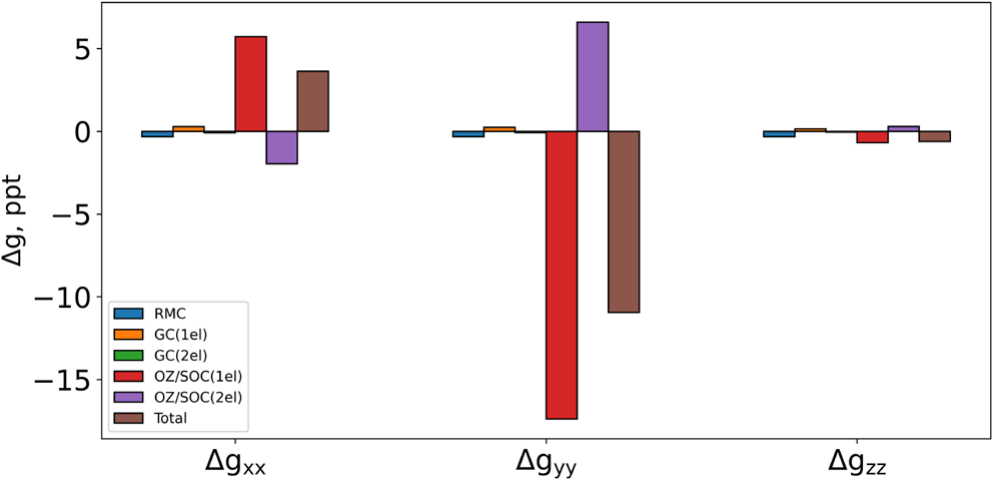
Contributions of RMC (relativistic mass correction), GC (gauge correction), and OZ/SOC (orbital Zeeman and spin–orbit), and total Δ*g* components for NO_2_ computed with CPKS at the B3LYP/def2-TZVP level.

### Heavy Atom Molecules

Next, we analyze a set of diatomic and triatomic molecules containing heavy atoms. The Δ*g* parameters obtained from CPKS, state-interaction TDDFT, and TDA calculations are presented in Table 2, together with available experimental data and two-component spin-polarized KS-DFT results reported by Malkin et al.,^48^ which were computed using the Douglas–Kroll–Hess (DKH) Hamiltonian and include scalar relativistic effects. These comparisons provide insight into the impact of incorporating SOC effects variationally via the more rigorous DKH Hamiltonian. However, caution is warranted when interpreting the results, as the underlying exchange–correlation functionals and basis sets differ from those employed in CPKS and state-interaction methods. Consistent with the trends observed for light diatomic molecules, the TDDFT approach predicts lower excitation energies than TDA, leading to larger computed *g*-shifts. Overall, the accuracy of DFT-based methods deteriorates with an increasing atomic number, leading to significant discrepancies, particularly for HgH and IrH_2_. This decline in performance is expected, as the BP Hamiltonian used in both the state-interaction and CPKS approaches is known to be inadequate for heavy elements.^43,95^

**Table 2:** Calculated Δ*g* Values (in ppt) for Di and Triatomic Molecules with CPKS, TDA, and TDDFT State-Interaction Methods^a^ at the B3LYP/ANO-RCC-TZVP Level

molecule	spin	*g*-shift	CPKS	TDA	TDDFT	2C-DKH^b^	exp.^c^
SeO	1	Δ*g*_∥_	0.0	–1.	–1.1	–0.7	
		Δ*g*_⊥_	18.8	9.4	9.7	2.2	19.3
NBr	1	Δ*g*_∥_	–0.1	–2.3	–2.4	–0.5	
		Δ*g*_⊥_	22.7	12.0	12.3	16.0	19.3
NI	1	Δ*g*_∥_	0.0	–23.7	–24.3	–1.2	
		Δ*g*_⊥_	54.8	3.2	3.2	20.3	31.0
PdH	1/2	Δ*g*_∥_	0.1	–21.6	–22.0	–16.5	–37.3
		Δ*g*_⊥_	139.8	122.8	124.1	224.2	290.6
CdH	1/2	Δ*g*_∥_	0.1	–3.1	–3.2	–1.9	–5.3
		Δ*g*_⊥_	–87.8	–99.4	–101.6	–59.8	–49.9
HgH	1/2	Δ*g*_∥_	0.2	–16.8	^d^	–25.9	–26.3
		Δ*g*_⊥_	340.2	341.2	^d^	–236.5	–174.0
RhH_2_	1/2	Δ*g*_*xx*_	–0.3	–119.9	–184.1		–0.3
		Δ*g*_*yy*_	576.8	505.9	598.8		679.1
		Δ*g*_*zz*_	568.7	493.2	610.6		860.9
IrH_2_	1/2	Δ*g*_*xx*_	0.3	–1088.5	^d^		–454.8
		Δ*g*_*yy*_	–332.2	–1358.8	^d^		661.7
		Δ*g*_*zz*_	3619.0	–827.7	^d^		1712.7

a Δ*g*_⊥_ obtained as the average of Δ*g*_*xx*_ and Δ*g*_*yy*_.

b Two-component Douglas–Kroll–Hess Hamiltonian using the BP86 functional and the uncontracted Hirao basis sets from ref 48.

c Experimental values taken from ref 40: (SeO, NBr, and NI), ref 57 (PdH, CdH, and HgH), ref 96 (RhH_2_ and IrH_2_).

d Unconverged calculation due to excited state instabilities.

Heteronuclear diatomic molecules belong to the *C*_∞*v*_ point group, and their response to an external magnetic field is characterized by two components: Δ*g*_∥_, aligned with the molecular axis, and doubly degenerate Δ*g*_⊥_, perpendicular to it. Across all studied molecules, the experimental Δ*g*_⊥_ consistently exhibits a magnitude larger than that of Δ*g*_∥_, a trend generally reproduced by the different DFT-based approaches. In SeO, both the state-interaction and 2C-DKH approaches significantly underestimate the experimental Δ*g*_⊥_ value, whereas CPKS shows excellent agreement. However, the reliability of the experimental value has been questioned.^48^ For the parallel component, CPKS yields 0.0 ppt, while both state-interaction and 2C-DKH methods predict values of around −1 ppt. For the halides NBr and NI, CPKS systematically overestimates the perpendicular *g*-shift, whereas TDDFT and TDA tend to underestimate it, with the latter trend being particularly pronounced in NI. Among all diatomic molecules analyzed, NI is unique in that its TDDFT/TDA-computed |Δ*g*_∥_| surpasses its perpendicular counterpart. For the three monohydrides investigated, both CPKS and state-interaction methods show notable deviations from experimental Δ*g*_⊥_ values. However, CPKS yields Δ*g*_⊥_ values that are slightly closer to experimental data despite underestimating this component in PdH and overestimating it in CdH and HgH. A key limitation of CPKS is its inability to capture the parallel component Δ*g*_∥_ in all diatomic molecules, consistently yielding near-zero values. In contrast, state-interaction methods produce sizable shifts, showing notably better agreement with experimental data for PdH, CdH, and HgH. However, despite this improvement, Δ*g*_∥_ remains systematically underestimated across all cases.

Triatomic molecules RhH_2_ and IrH_2_ belong to the *C*_2*v*_ point group. In the following, we align the *z*-axis with the 2-fold rotation axis, the *y*-axis within the molecular plane, and the *x*-axis perpendicular to it. For RhH_2_, both CPKS and state-interaction approaches yield comparable in-plane shifts, Δ*g*_*yy*_ and Δ*g*_*zz*_, which are of similar magnitudes but systematically smaller than experimental values. In contrast, for IrH_2_, both CPKS and TDDFT/TDA exhibit significant deviations from the experimental in-plane shifts. Consistent with its performance for the parallel component in diatomic molecules, CPKS predicts a very small Δ*g*_*xx*_ for both triatomic molecules. While this result agrees well with experimental data for RhH_2_, it deviates significantly from the measured value for IrH_2_. On the other hand, TDDFT and TDA yield significantly larger parallel shifts in both molecules, though in neither case do the computed values fully align with the experiment.

To better understand and rationalize these results, we analyze the contribution of individual nonrelativistic excited states to the overall *g*-shifts. For this purpose, we compute Δ*g* values for NI, PdH, and HgH using two-state SOC-dressed Hamiltonians, incorporating the sublevels of the KS ground state and a single TDA root (Figure 3). This approach is conceptually similar to evaluating individual terms in an SOS expansion. However, by explicitly diagonalizing the effective Hamiltonian that couples the ground and excited states and mapping eqs 1 and 2 in the basis of the ground eigenstate multiplet, our computed *g*-parameters capture not only first-order effects but also second-order contributions. Based on the number of nonrelativistic excited states that significantly contribute to Δ*g*, the studied molecules can be classified into two distinct categories: those influenced by multiple excited states and those dominated by a single excited state per direction.

**Figure 3:**
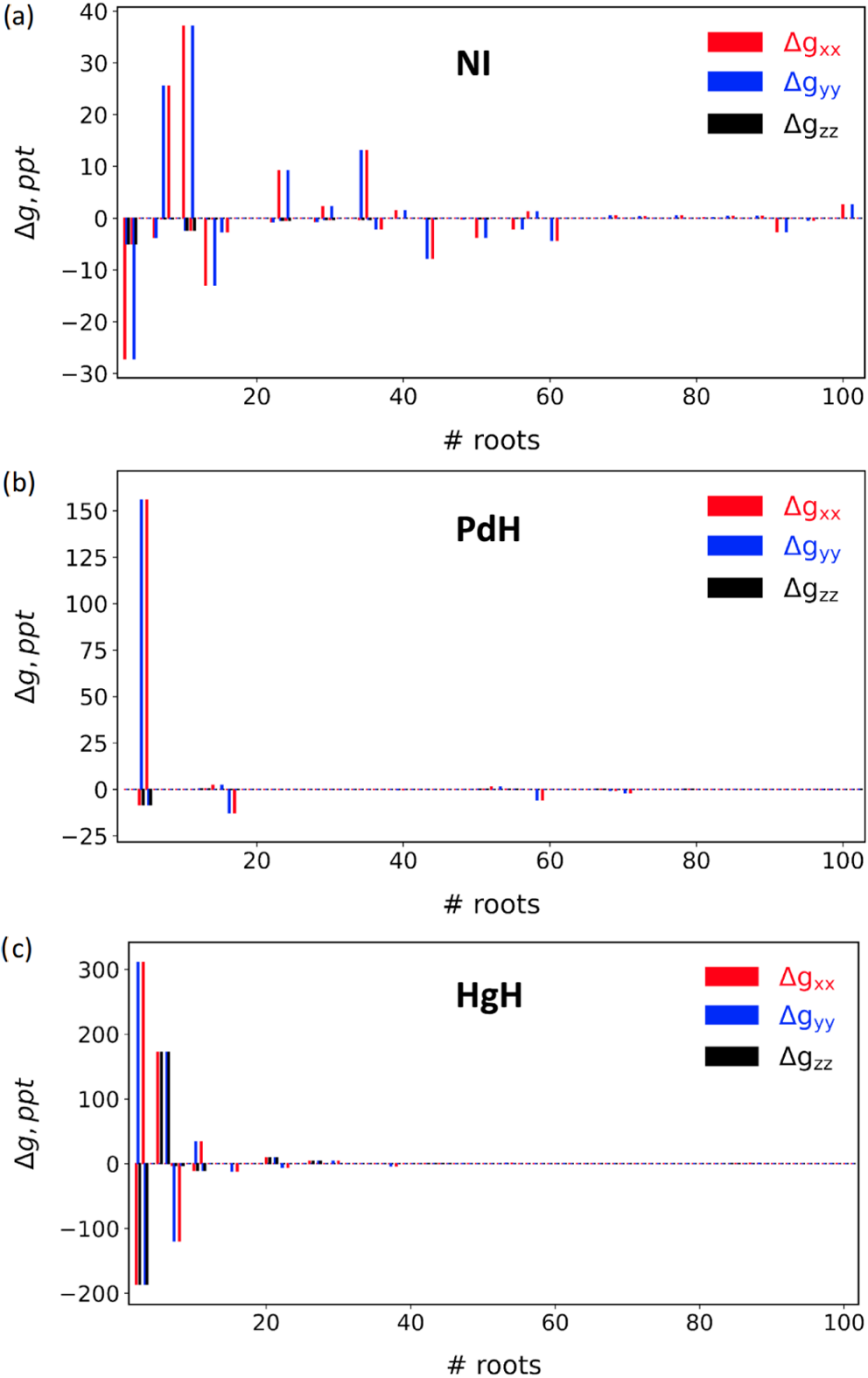
Δ*g* components obtained with two-state SOC-dressed Hamiltonians, the ground and a single excited state, for NI, PdH, and HgH molecules. Perpendicular (Δ*g*_⊥_) components: Δ*g*_*xx*_ and Δ*g*_*yy*_. Parallel (Δ*g*_∥_) component: Δ*g*_*zz*_.

First, in all studied cases, individual state contributions decrease as the excitation energy increases, becoming nearly negligible for the highest-energy excitations (approaching state number 100). This trend indicates the convergence of our state-interaction approach with respect to the number of included states, as shown for the NBr and SeO cases in Table S8.

The main contributions to the Δ*g* terms in SeO emerge from the lowest excited states (Figure S12). In the nitrogen halides (Figures 3a and S13), multiple electronic excited states significantly contribute to the *g*-shifts, particularly to the perpendicular components. In both molecules, the dominant contributions to Δ*g*_⊥_ arise from the strong SOC between the ^3^Σ^–^ ground state and the doubly degenerate ^3^Π excited states. This is consistent with symmetry selection rules derived from the perturbative expression used to evaluate Zeeman and spin–orbit contributions^69,97^

12 $$\Delta g_{\mathrm{ki}}=\frac{-2}{S}\sum_{I\neq 0}\frac{\left\langle 0\right|L_{k}\left|I\right\rangle \left\langle I\right|h_{i}^{\mathrm{SO}}\left|0\right\rangle }{E_{I}-E_{0}};,k,i=x,y,z$$

where *S* is the spin of the ground state 0 with *E*_0_ energy, {*I*} are the nonrelativistic excited states with {*E*_*I*_} energies, *h*_*i*_^SO^ is the *i*th Cartesian component of the mean-field SOC operator,^64^ and *L*_*k*_ is the *k*th component of the orbital angular momentum operator. Interestingly, while symmetry considerations based on eq 12 suggest that ^3^Π states should not contribute to Δ*g*_∥_, our calculations reveal that they do. This implies that for Δ*g*_∥_, the participation of ^3^Π states occurs as a second-order SOC effect. Such an interpretation aligns with the near-zero Δ*g*_∥_ values obtained using CPKS, which inherently lack these second-order contributions.

The single-state analysis for the three studied monohydrides, PdH, CdH, and HgH, reveals that only one or a few states significantly contribute to the TDA-computed *g*-parameters, as shown in Figure 3b,c for PdH and HgH, respectively. Similar to the case for NBr and NI, the perpendicular components in the monohydrides arise from the first-order, symmetry-allowed coupling of the X^2^Σ^+^ ground state with the lowest-lying ^2^Π excitations. These same ^2^Π states also contribute to Δ*g*_∥_, despite being symmetry-forbidden in first-order SOC interactions, as previously discussed for nitrogen halides. This aligns with the near-zero Δ*g*_∥_ values obtained by using CPKS.

The influence of second- and higher-order SOC effects on *g*-parameters, as anticipated by our symmetry-based analysis of single-state contributions, has been previously discussed in the literature. Patchkovskii and Ziegler attributed the significant error in the computed *g*-shift of NI to the neglect of higher-order SOC effects.^40^ Similarly, Lan et al. demonstrated that first-order approaches are insufficient for accurately describing Δ*g* in the hydrides and dihydrides examined in this study, highlighting the necessity of incorporating higher-order SOC effects. Malkin et al. highlights the importance of higher-order SOC effects in both the parallel and perpendicular values of these molecules.^48^ To further investigate the impact of different SOC orders, we analyze the dependence of state-interaction-computed Δ*g* on the scaling of SOC interactions.^48,57^ Specifically, we calculate Δ*g* by applying our state-interaction procedure with a scaled effective Hamiltonian, where the off-diagonal SOC elements are modified as

13 $$H_{\mathrm{IJ}}^{\mathrm{eff}}=E_{I}\delta_{\mathrm{IJ}}+\lambda H_{\mathrm{IJ}}^{\mathrm{SO}}$$

Here, λ is a scaling factor that modulates the strength of the SOC contribution. Table 3 presents the coefficients obtained by fitting Δ*g* as a function of the SOC scaling factor to a third-order polynomial for the diatomic molecules. Equivalent analysis for RhH_2_ and IrH_2_ can be found in the Supporting Information (Table S5).

**Table 3:** Fitted Parameters (in ppt) for the Dependence of the Perpendicular and Parallel *g*-Shifts on the SOC Scaling Factor in Diatomic Molecules^a^

molecule	*Z*_max_	*c*_3_	*c*_2_	*c*_1_	*c*_0_
Δ*g*_⊥_ Component					
SeO	34	0.00	–1.67	11.03	0.00
NBr	35	–0.03	–2.43	14.50	0.00
NI	53	1.51	–27.50	29.18	–0.02
PdH	46	–0.19	–15.98	138.91	0.00
CdH	48	–0.13	–6.40	–92.86	0.00
HgH	80	–76.11	181.52	236.07	–0.25
Δ*g*_∥_ Component					
SeO	34	0.02	–1.07	0.00	0.00
NBr	35	0.02	–2.33	0.01	0.00
NI	53	2.51	–26.70	0.55	–0.02
PdH	46	2.42	–24.02	0.04	0.00
CdH	48	–0.28	–2.81	0.00	0.00
HgH	80	–23.80	–7.72	15.70	–0.63

a The *g*-shift is modeled as Δ*g* = *c*_3_λ^3^ + *c*_2_λ^2^ + *c*_1_λ + *c*_0_, where λ is the SOC scaling parameter, and Δ*g* and the coefficients *c*_*i*_ (*i* = 0,3) are given in ppt. For all molecules, the fit achieves an accuracy of *R* ^2^ = 1.0. *Z*_max_ indicates the atomic number of the heaviest element.

The perpendicular component in all diatomic molecules listed in Table 3 is primarily governed by the linear SOC term (*c*_1_ coefficient). However, the contributions from second- and third-order SOC increase with atomic number, with second-order effects reaching a magnitude comparable to the first-order term in NI and HgH and third-order effects becoming significant in HgH. Interestingly, the small Δ*g*_⊥_ value obtained for NI using the state-interaction approach arises from a cancellation between first- and second-order contributions. A comparison with 2C-DKH results^48^ suggests that our calculations, based on the BP spin–orbit Hamiltonian, tend to overestimate the magnitude of the second-order term. In SeO, the situation is reversed: the second-order SOC contribution nearly cancels the first-order term in the 2C-DKH results (*c*_1_ = 15.8 ppt and *c*_2_ = −13.4 ppt),^48^ leading to a small net Δ*g*_⊥_. However, the second-order contribution obtained from our state-interaction calculations is significantly smaller, resulting in a less pronounced cancellation.

In contrast, Δ*g*_∥_ is predominantly determined by second-order terms, consistent with the first-order symmetry-based selection rules discussed earlier and the vanishing values obtained with CPKS. Interestingly, the linear term is relatively large for the parallel shift in HgH, although this particular fit may not be reliable. The independent term (*c*_0_) serves as an indicator of the accuracy of the third-order polynomial fit. Ideally, in the absence of SOC (λ = 0) and other relativistic effects, Δ*g* should vanish. This condition holds for all molecules in Table 3 except for HgH, which contains a heavy Hg atom (*Z* = 80). These findings suggest that even higher-order SOC effects contribute to the state-interaction TDA Δ*g* values, particularly in systems with heavy elements, such as Hg.

To further elucidate the origin of the *g*-parameters, we combine the single-state analysis with the SOC scaling approach. Specifically, we investigated the contributions of the angular momentum and spin operators to the Δ*g* components. For this purpose, we analyze the perpendicular and parallel *g*-shifts in NI, derived from a two-state effective Hamiltonian that includes the ground X^3^Σ^–^ state and the lowest ^3^Π excited state. Figure 4 reveals a perfect linear dependence of Δ*g*_⊥_ on the SOC scaling factor. Furthermore, when the angular momentum operator is removed from the Zeeman Hamiltonian (eq 1), the shift becomes zero, regardless of the SOC scaling. This indicates that the contribution from the lowest ^3^Π state to Δ*g*_⊥_ is linear in the SOC, mediated by the orbital Zeeman operator. In contrast, the two-state Δ*g*_∥_ shows a quadratic dependence on the SOC scaling factor. Interestingly, this profile remains unchanged when the angular momentum operator is removed, indicating that the parallel component is a second-order SOC effect mediated through the spin operator.

**Figure 4:**
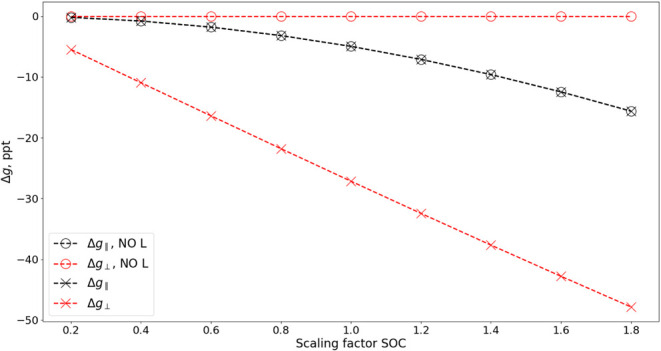
Profiles of Δ*g*_∥_ (black) and Δ*g*_⊥_ (red) as functions of the SOC scaling factor computed with the X^3^Σ^–^ and ^3^Π SOC-dressed Hamiltonian for the NI molecule. The results are shown with (crosses) and without (empty circles, “NO L”) inclusion of the angular momentum operator in the Zeeman Hamiltonian.

### Transition-Metal Complexes

In this section, we assess the performance of the CPKS and state-interaction TDDFT/TDA approaches in predicting *g*-values of transition-metal complexes: TiF_3_, [Ni­(mnt)_2_]^−^, [CuCl_4_]^2–^, [Cu­(NH_3_)_4_]^2+^, and [MOX_4_]^*n*−^ with M: V, Cr, Mo, and X: F, Cl, and Br. Table 4 presents the computed *g*-shifts for the investigated transition-metal complexes. TDDFT/TDA results are computed using 500 states (analysis of the dependence of computed *g*-parameters with the number of states can be found in the Supporting Information). As in the heavy atom molecules, in the TDDFT state-interaction procedure Δ*g*_⊥_ is computed as the average of Δ*g*_*xx*_ and Δ*g*_*yy*_. Overall, the performance of state-interaction TDDFT and TDA is comparable to that of the CPKS method, exhibiting similar trends across the studied systems. A comparison of *g*-shifts computed using TDA and CPKS with the B3LYP and BLYP exchange–correlation functionals is provided in the Supporting Information (Table S7). In general, both functionals exhibit similar trends; however, as noted in previous studies,^30,37^ the dependence on the functional is more pronounced for these systems than for light-atom molecules (Figure 1).

**Table 4:** Calculated Δ*g* Values (in ppt) for Transition-Metal Complexes Molecules Using in CPKS, TDA and Full TDDFT, the B3LYP Functional and the def2-TZVP Basis Set

molecule		CPKS	TDA	TDDFT	exp.^a^
TiF_3_	Δ*g*_∥_	–1.3	15.8	15.7	–11.1, −3.72
	Δ*g*_⊥_	–46.1	–57.8	–61.4	–111.3, −123.72
[Ni(mnt)_2_]^−^	Δ*g*_*xx*_	103.4	72.0	74.5	157.7
	Δ*g*_*yy*_	62.5	17.8	18.1	39.7
	Δ*g*_*zz*_	–5.5	–18.6	–18.6	–4.3
[CuCl_4_]^2–^	Δ*g*_∥_	141.6	162.4	165.6	230.3
	Δ*g*_⊥_	40.0	23.8	23.9	46.7
[Cu(NH_3_)_4_]^2+^	Δ*g*_∥_	146.8	166.9	169.9	238.7
	Δ*g*_⊥_	40.3	30.1	30.5	44.7
[VOF_4_]^2–^	Δ*g*_∥_	–41.4	–53.2	–54.5	–70.5
	Δ*g*_⊥_	–27.9	–18.6	–19.6	–30.5
[VOCl_4_]^2–^	Δ*g*_∥_	–22.3	–24.5	–25.7	–54.5
	Δ*g*_⊥_	–22.1	–15.7	–16.3	–23.3
[VOBr_4_]^2–^	Δ*g*_∥_	56.5	43.6	44.3	
	Δ*g*_⊥_	–21.9	2.9	2.2	
[CrOF_4_]^−^	Δ*g*_∥_	–22.4	–33.1	–33.7	–43.3
	Δ*g*_⊥_	–31.1	–10.1	–11.0	–34.3
[CrOCl_4_]^−^	Δ*g*_∥_	17.71	–3.8	–3.8	–13.3
	Δ*g*_⊥_	–26.2	–4.3	–4.8	–26.3
[CrOBr_4_]^−^	Δ*g*_∥_	120.5	93.7	94.1	
	Δ*g*_⊥_	–32.2	–8.2	–8.3	
[MoOF_4_]^−^	Δ*g*_∥_	7.1	5.5	5.5	–107.5
	Δ*g*_⊥_	–2.8	–0.5	–0.5	–77.0
[MoOCl_4_]^−^	Δ*g*_∥_	28.9	21.3	21.5	–37.3
	Δ*g*_⊥_	–1.0	5.1	5.1	–56.1
[MoOBr_4_]^−^	Δ*g*_∥_	128.6	94.0	94.8	
	Δ*g*_⊥_	4.9	50.3	50.1	

a Experimental values from ref 98 [CuCl_4_]^2–^, ref 99 [Cu­(NH_3_)_4_]^2+^, ref 100 TiF_3_, ref 101 [VOF_4_]^2–^, [MoOF_4_]^−^, [MoOCl_4_]^−^, ref 102 [VOCl_4_]^2–^, ref 103 [CrOF_4_]^−^, ref 104 [CrOCl_4_]^−^, ref 105 [Ni­(mnt)_2_]^−^.

### Titanium­(III) Fluoride

The TiF_3_ molecule adopts a planar (*D*_3*h*_) geometry with a spin-doublet ground state, ^2^A′_1_, characterized by an unpaired electron predominantly localized in the 3d_*z* ^2^_ orbital of Ti. As expected from the significant ionic character of the Ti–Cl bonding,^26^ the dominant contribution to Δ*g*_⊥_ arises from the first-order interaction between the ground state and the 2-fold-degenerate ligand-field state 1^2^E″, where the unpaired electron occupies either the 3d_*xz*_ or 3d_*yz*_ orbital of the metal. This interaction is particularly relevant due to the relatively small energy gap (Δ*E* = 0.966 eV) and significant SOC constant (SOCC = 123 cm^–1^). The computed Δ*g*_⊥_ values at the TDA and TDDFT levels underestimate the magnitude of the experimental shift but show notable improvement over the CPKS result. This discrepancy can largely be attributed to an overestimation of the excitation energy to 1^2^E″. Replacing the TDA/TDDFT excitation energies with the CASPT2 value reported by Vancoillie et al.^26^ (Δ*E* = 0.594 eV) causes the computed Δ*g*_⊥_ to shift significantly closer to the experimental value (Δ*g*_⊥_ = −96.2 ppt). In contrast, while the Δ*g*_∥_ calculated with CPKS shows a significant deviation from the experimental value, in this molecule, both TDDFT and TDA yield shifts of comparable magnitude but with opposite signs. The state-interaction approach identifies a high-energy (Δ*E* = 10.144 eV) excited doublet belonging to the A′_2_ irreducible representation as a first-order contributor to Δ*g*_∥_ (SOCC = 190 cm^–1^), while a second-order contribution primarily originates from the 1^2^E″ excitation, consistent with wave function-based calculations.^20^

### Bis­(maleonitriledithiolato)­nickelate­(III) Complex

The [Ni­(mnt)_2_]^−^ complex, where mnt denotes the maleonitriledithiolato bidentate ligand, adopts *D*_2*h*_ symmetry. For consistency, we consider the molecule oriented in the *xy*-plane, with the ligand’s carbon–carbon bond aligned along the *x*-direction. It features a spin-doublet ground state with a single unpaired electron occupying a b_3g_ orbital (X^2^B_3g_). This orbital is predominantly composed of the metal-centered 3d_*yz*_, with significant contributions from the ligands’ *p*_*z*_ orbitals, as reflected in the spin-density distribution (Figure 5).

**Figure 5:**
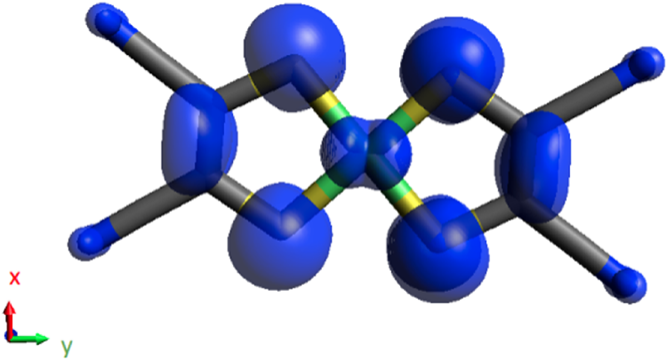
Spin-density distribution (isovalue of 0.0008 *ea*_0_^–3^) of the [Ni­(mnt)_2_]^−^ doublet ground state computed at the B3LYP/def2-TZVP level.

The *g*-matrix component perpendicular to the molecular plane (Δ*g*_*zz*_) computed using both CPKS and state-interaction approaches shows good agreement with experimental measurements. In contrast, the underestimation of Δ*g*_*xx*_ observed in CPKS is even more pronounced in TDA and TDDFT. While CPKS overestimates the shift along the *y*-direction, TDA and TDDFT underestimate it, though with smaller deviations from the experimental values. Single-state analysis reveals contributions from multiple excited states to each Δ*g* component (Figure S15). These contributions primarily arise from first-order interactions between the ground state and excited states of different symmetries: ^2^A_g_ for Δ*g*_*xx*_, ^2^B_1g_ for Δ*g*_*yy*_, and ^2^B_2g_ for Δ*g*_*zz*_.

Previous studies have shown that in [Ni­(mnt)_2_]^−^, sulfur nuclei contribute exclusively to Δ*g*_*xx*_, while Δ*g*_*yy*_ and Δ*g*_*zz*_ arise purely from SOC effects induced by Ni electrons.^106^ The strong covalency of the Ni–S bond in [Ni­(mnt)_2_]^−^ leads to spin delocalization, significantly impacting the *g*-shifts.^89,106^ Accurately computing Δ*g* values thus requires a proper description of the spin-density distribution between the transition-metal and its ligands. Pure DFT functionals tend to overestimate metal–ligand bond covalency, which can lead to inaccurate predictions. Hybrid functionals, incorporating a fraction of HF exchange (HFX), can mitigate this issue.^32^ However, while increasing HFX content may improve accuracy in some complexes, it can also introduce spin contamination and deteriorate results.^70^ This trend is reflected in Table S2, which shows that spin contamination is consistently lower for all molecules when using the pure GGA functional BLYP compared to the hybrid B3LYP functional, consistent with the findings of Menon et al.^107^

To assess the impact of HFX on the computed *g*-parameters of [Ni­(mnt)_2_]^−^, we perform state-interaction TDA calculations using B3LYP as the reference functional (HFX percentage: 20%) and systematically vary the HFX fraction (Table 5). As expected, increasing the HFX fraction reduces the spin population on the central Ni atom, indicating greater metal–ligand covalency. This leads to a decrease in Δ*g*_*xx*_, while Δ*g*_*yy*_ and Δ*g*_*zz*_ generally increase, although the trend for Δ*g*_*zz*_ is less systematic.

**Table 5:** Δ*g* Components (in ppt) and Ground-State Mulliken Spin Population of the Ni Atom in [Ni­(mnt)_2_]^−^ Obtained with B3LYP-like Functionals with Different Percentages of HFX^a^

					1^2^A_2g_				X^2^B_3g_
% HFX	Δ*g*_*xx*_	Δ*g*_*yy*_	Δ*g*_*zz*_	Ni spin pop.	Δ*E*	SOCC	Δ*g*_*xx*_	⟨*Ŝ* ^2^⟩	⟨*Ŝ* ^2^⟩
10	89.0	9.5	11.8	0.28	1.194	392	69.3	0.757	0.755
15	74.3	12.7	9.7	0.28	1.186	393	68.3	0.760	0.757
20	71.7	23.0	–4.0	0.27	1.211	386	62.0	0.764	0.760
25	58.2	26.5	–4.7	0.24	1.297	361	47.4	0.771	0.764
30	35.4	42.2	11.6	0.18	1.493	345	30.1	0.774	0.768
35	11.0	41.1	37.3	0.11	1.813	277	10.0	0.781	0.773
40	–3.3	39.3	4.6	0.05	2.222	210	11.6	0.800	0.780
exp.	158^b^	40^b^	–4^b^	0.32^c^					

a Excitation energies (in eV), SOCC (in cm^–1^), Δ*g*_*xx*_ and ⟨*Ŝ* ^2^⟩ of the excited state 1^2^A_2g_, and ground state X^2^B_3g_ ⟨*Ŝ*_2_⟩.

b From ref 98.

c From ref 108.

The dependence of the *g*-shifts on HFX can be further analyzed through single-state contributions. Table 5 also reports the excitation energy and SOCC for the excited state with the largest contribution to Δ*g*_*xx*_, namely 1^2^A_2g_. This state primarily arises from an electron excitation from a doubly occupied orbital, with significant involvement of the metal’s 3d_*z* ^2^_ orbital, to the b_3g_-SOMO, accounting for the observed positive Δ*g*_*xx*_ value.^69^ The computed *g*-shift from this state closely approximates the total TDA value (except for 40% HFX), confirming that its coupling with the ground state predominantly determines the Δ*g*_*xx*_. As the HFX fraction increases, the excitation energy rises, while the SOCC decreases, both contributing to the observed trend in Δ*g*_*xx*_.

The effect of spin contamination across different families of functionals has been previously investigated, for example, in the context of magnetic interactions by Kaupp et al.,^70,109^ and in comparisons between unrestricted and restricted treatments by Menon and Radom.^107^ These studies concluded that the inclusion of exact exchange in hybrid functionals tends to increase spin contamination compared with pure DFT functionals. In our case, increasing the HFX fraction similarly leads to greater spin contamination in both the ground (X^2^B_3g_) and excited (1^2^A_2g_) states. However, as shown in Table 5, the computed ⟨*Ŝ* ^2^⟩ values across the entire explored HFX range (10–40%) remain reasonably close to the ideal value of 0.75 for a pure spin doublet.

### Cu­(II) Complexes

The magnetic properties of the copper complexes [CuCl_4_]^2–^ and [Cu­(NH_3_)_4_]^2+^, particularly the anisotropy of the *g*-matrix, have been investigated computationally at various levels of theory.^26,30,69,110^ Both compounds feature a tetracoordinated Cu^2+^ cation with a *d* ^9^ electronic configuration and a square-planar coordination environment. While [CuCl_4_]^2–^ formally belongs to the *D*_4*h*_ symmetry point group, the presence of hydrogen atoms in [Cu­(NH_3_)_4_]^2+^ reduces its symmetry to *D*_2*d*_. However, since hydrogen atoms have a negligible effect on the ground-state spin density (Figure S20) and the description of low-lying electronic transitions, both complexes are analyzed within the *D*_4*h*_ framework. In this convention, the metal and coordinating atoms lie in the *xy*-plane, with the *x*- and *y*-axes aligned along the Cu–X bonds (X = Cl, N).

CPKS and state-interaction TDDFT/TDA yield comparable results for both Cu complexes (Table 4), consistently underestimating the values of Δ*g*_∥_ and Δ*g*_⊥_. While CPKS provides a more accurate prediction of Δ*g*_⊥_, TDDFT and TDA better capture Δ*g*_∥_. The ground state of both compounds corresponds to the X^2^B_1g_ state, with an unpaired electron primarily localized in the metal’s 3d_*x* ^2^–*y* ^2^_ orbital and significant covalent delocalization. Previous studies have rationalized the *g*-values of these complexes using ligand-field models that relate bonding ionicity to *g*-shifts,^27,30,32,69^ where greater ionic character leads to larger shifts. Mulliken analysis of the X^2^B_1*g*_ state at the B3LYP level yields Cu spin populations of 0.49 and 0.57 for [CuCl_4_]^2–^ and [Cu­(NH_3_)_4_]^2+^, respectively, significantly lower than values obtained from highly correlated methods.^111^ In contrast, the CASSCF wave function exaggerates spin localization on the metal, leading to a systematic overestimation of *g*-values.^111^

Analysis of the state-interaction results for both compounds reveals that each component of the *g*-matrix is primarily determined by the first-order effect of a single excited state, as illustrated in the decomposition of individual state contributions for [Cu­(NH_3_)_4_]^2+^ (Figure S17). The Δ*g*_∥_ shift arises from the interaction between the ground state and the lowest ^2^B_2g_ excited state, with a TDDFT (TDA) computed excitation energy of 2.53 (2.57) eV and a SOCC of 970 cm^–1^. The underestimation of Δ*g*_∥_ by TDDFT and TDA can be attributed to an overestimated excitation energy for ^2^B_2g_. Substituting the computed value with the experimental transition energy (Δ*E* = 1.74 eV^32^) increases the predicted *g*-shift to 258.5 ppt, closer to the experimental value (238.7 ppt). The remaining discrepancy with the experiment likely stems from inaccuracies in spin–orbit interactions, as previously suggested by Neese within a ligand-field sum-over-states framework.^30^ The dominant contribution to Δ*g*_⊥_ comes from the interaction with the lowest 2-fold-degenerate ^2^E_*g*_ excited state. Similar to the case for Δ*g*_∥_, the underestimation of Δ*g*_⊥_ can be linked to inaccuracies in excitation energies. TDDFT predicts Δ*E* = 2.58 eV, exceeding the experimental value of Δ*E* = 2.17 eV.^32^

### [MOX_4_]^*n*−^ Complexes

Since the [MOX_4_]^*n*−^ series (M = V, Cr, Mo; X = F, Cl, Br) belongs to the *C*_4*v*_ symmetry point group, the Δ*g* parameters can be decomposed into Δ*g*_∥_, oriented along the M–O bond (*z*-axis), and Δ*g*_⊥_, which accounts for the doubly degenerate components in the *xy*-plane. Overall, DFT-based results for [MOX_4_]^*n*−^ complexes with first-row transition metals (M = V, Cr) show good agreement with experimental data. However, for [MoOX_4_]^−^, the computed *g*-shifts deviate significantly from the available experimental values, underscoring the limitations of these approaches. For vanadium and chromium compounds, CPKS and state-interaction approaches yield similar results, with CPKS providing Δ*g*_⊥_ values closer to the experiment, while state-interaction methods perform better for the Δ*g*_∥_ component. The analysis of individual *g*-shift contributions for [VOF_4_]^2−^, [VOCl_4_]^2−^, and [CrOF_4_]^−^ (Figures 6, S18, and S19) indicates that convergence is achieved with 500 excited states. In contrast, for the remaining molecules, significant contributions from high-energy excited states suggest that the *g*-shift calculations may be affected by an insufficient number of states, potentially compromising accuracy.

**Figure 6:**
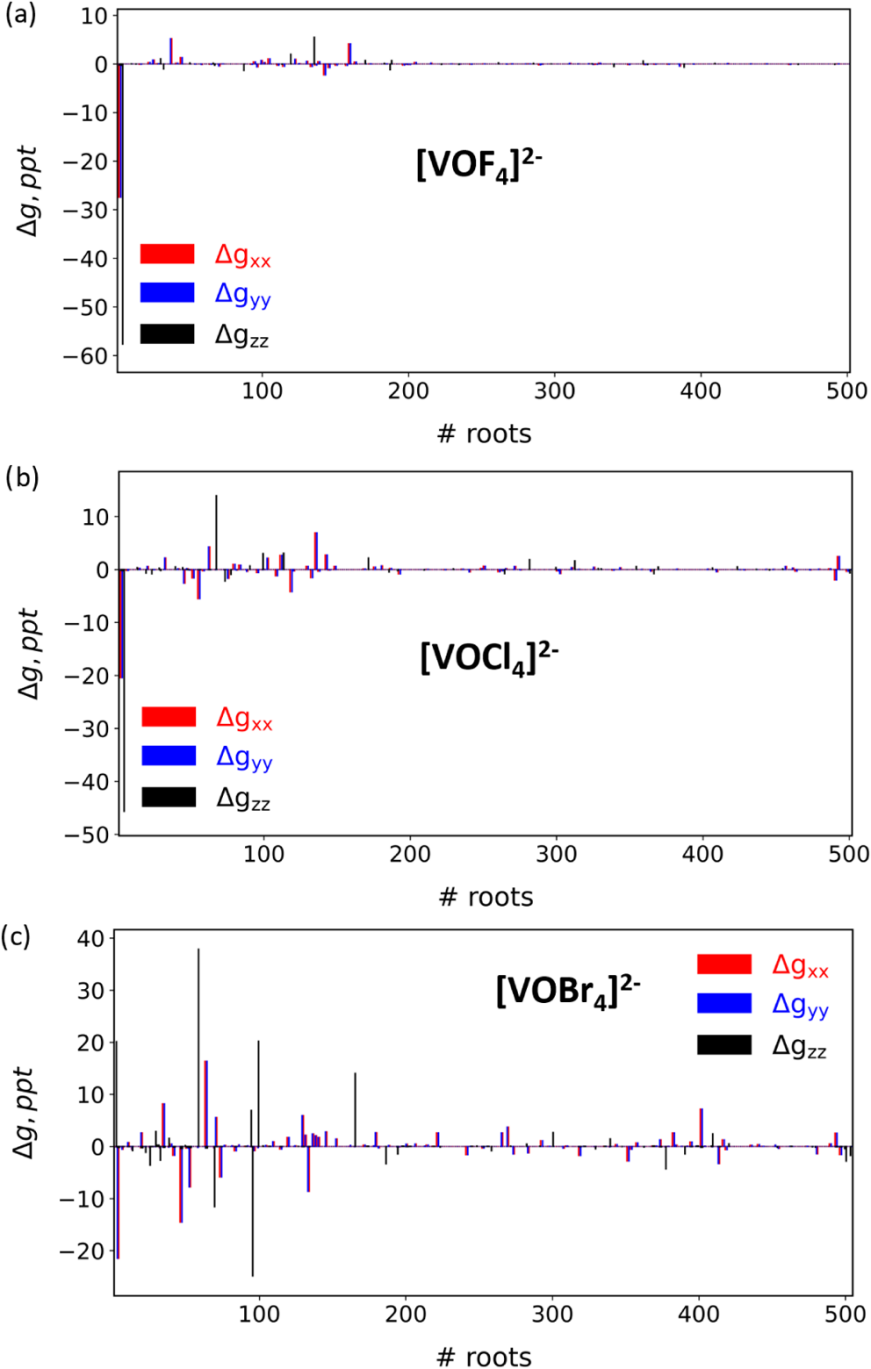
Individual state contribution to Δ*g*_∥_ (Δ*g*_*zz*_) and Δ*g*_⊥_ (Δ*g*_*xx*_ and Δ*g*_*yy*_) in two-state SOC-dressed Hamiltonians, the ground and a single excited state (# roots in the *x*-axis) for [VOX_4_]^2–^, with X= F, Cl, Br.

Finally, we examine the ligand’s influence by analyzing single-state contributions, focusing on the [VOX_4_]^2–^ complexes (Figure 6). In all cases, the perpendicular *g*-shifts arise from the coupling between the X^2^B_2_ ground state, where the unpaired electron is primarily localized on the metal’s *d*_*xy*_ orbital, and doubly degenerate ^2^E excited states. Meanwhile, Δ*g*_∥_ originates from interactions with the ^2^B_1_ excitations. For fluorine ligands, only one excited state significantly contributes to each Δ*g* component. As the halogen atomic number increases, additional excited states become relevant. Notably, in [VOBr_4_]^2–^, a large number of states contribute across different directions, with high-lying excitations playing a crucial role, particularly in Δ*g*_*zz*_. Similar trends are observed for Cr (Figure S18) and Mo complexes (Figure S19).

### Role of Excited State Spin Contamination

Throughout this paper, we have shown that CPKS and state-interaction TDDFT/TDA methodologies generally yield comparable *g*-parameters, particularly for molecules composed of light atoms. However, in several cases, notable discrepancies are observed between the two approaches. Examples include Δ*g*_⊥_ in NBr, Δ*g*_∥_ in TiF_3_, and all of the Δ*g* components in [CrOF_4_]^−^ and [CrOCl_4_]^−^. As discussed previously, one possible origin of these discrepancies is the contribution of second- and higher-order SOC effects, which are not captured by the CPKS approach. Nonetheless, our SOC scaling analysis (Table 3) indicates that the linear SOC contribution is significantly larger than higher-order terms in these cases, suggesting that this is not the primary cause of the observed differences. Another possible explanation is insufficient convergence in the state-interaction calculations due to a limited number of excited states. To examine this, we evaluated the dependence of computed *g*-shifts on the number of excited states included in the effective Hamiltonian. As shown in Table S8, results obtained using 100, 300, and 500 excited states confirm that convergence is achieved for NBr, TiF_3_, [CrOF_4_]^−^, and [CrOCl_4_]^−^, ruling this factor out as the source of the discrepancy.

We next explored the potential influence of spin contamination in excited states obtained via TDDFT/TDA. To diagnose this effect, we introduce a quantity that captures the contribution of spin-contaminated excited states to the computed *g*-shifts

14 $$\sigma_{k}=\left(\langle {Ŝ}^{2}\rangle_{\mathrm{calc}}-\langle {Ŝ}^{2}\rangle_{\mathrm{ideal}}\right)\cdot \Delta g_{\mathrm{kk}},,\mathrm{for}\;k=x,y,z$$

Here, σ_*k*_ takes nonzero values for excited states that both deviate from the ideal spin value and contribute significantly to Δ*g*_*kk*_. Figure 7 displays the individual σ_*k*_ values for NBr and TiF_3_. Interestingly, for NBr (Figure 7a), we identify several spin-contaminated states that contribute significantly to Δ*g*_⊥_, while contributions to Δ*g*_∥_, for which TDA and CPKS are in better agreement, are negligible. In contrast, for TiF_3_ (Figure 7b), prominent spin-contaminated contributions are found for Δ*g*_∥_, with a minimal impact on the perpendicular components. A similar σ_*k*_ analysis for [CrOF_4_]^−^ and [CrOCl_4_]^−^ also reveals the presence of spin-contaminated excited states contributing to the *g*-shifts, further supporting the hypothesis that spin contamination may underlie discrepancies between CPKS and TDDFT/TDA results.

**Figure 7:**
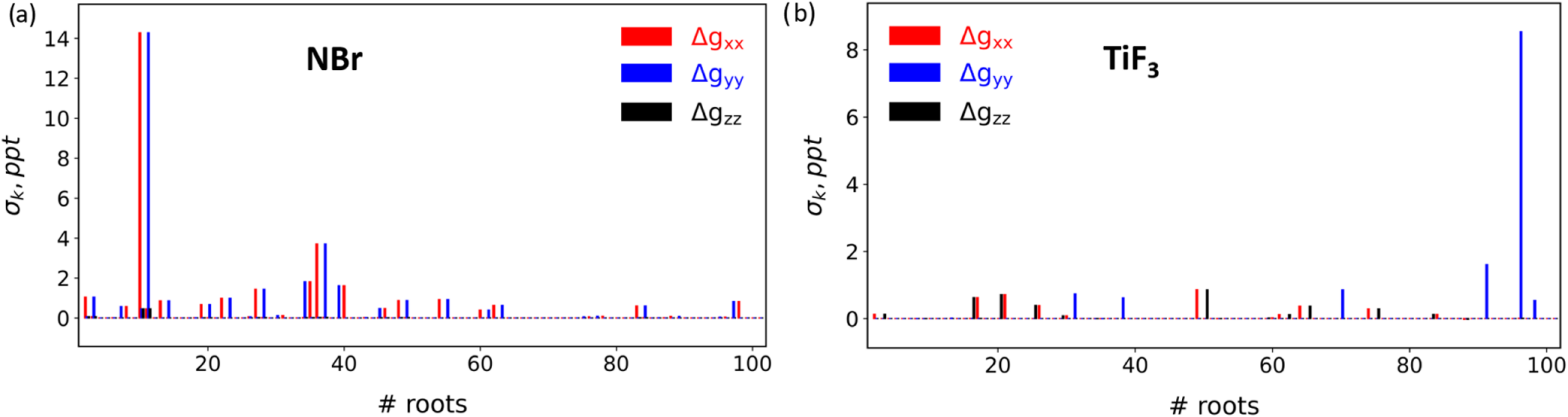
Diagnostic function (σ_*k*_ in ppt) identifying spin-contaminated states contributing to *g*-shifts in (a) NBr (Δ*g*_*z*_ ≡ Δ*g*_∥_; Δ*g*_*x*,*y*_ ≡ Δ*g*_⊥_) and (b) TiF_3_ (Δ*g*_*y*_ ≡ Δ*g*_∥_; Δ*g*_*x*,*z*_ ≡ Δ*g*_⊥_).

These observations suggest that spin contamination in excited states can play a significant role in the accuracy of *g*-shifts computed via the state-interaction approach. While this analysis provides evidence pointing toward this conclusion, it does not constitute definitive proof. Further investigation is necessary to fully understand the impact of spin contamination on the reliability of state-interaction-derived *g*-parameters.

### Computational Efficiency

The state-interaction TDDFT/TDA approach offers advantages over CPKS for computing *g*-matrices, including the inclusion of second- and higher-order SOC effects and the ability to characterize contributions within a nonrelativistic state basis. However, these benefits come at the cost of increased computational expense. While a comprehensive benchmarking of computational times is beyond the scope of this work, we explicitly compare the computational cost of the studied molecules. Table 6 reports CPU time data for CPKS and state-interaction TDA calculations performed with the B3LYP functional and the def2-TZVP basis set. All calculations were carried out using 6 cores on a single node equipped with Intel Xeon Gold 6342 processors. The reported times demonstrate the superior efficiency of CPKS, with state-interaction TDA calculations requiring approximately 3 to 14 times more computational time.

**Table 6:** Central Processing Unit (CPU) Times (in Seconds) to Perform CPKS and TDA Calculations Using def2-TZVP Basis Set for the Set of Studied Compounds^a^

		CPU times		
molecule	no. basis	CPKS	TDA	ratio
H_2_O^+^	43	3.2	12.6	3.9
CO_2_^–^	93	11.5	93.0	8.1
NO_2_	93	11.2	87.0	7.8
TiF_3_	138	35.7	333.6	9.3
[CuCl_4_]^2–^	193	126.5	879.4	7.0
[MoOF_4_]^−^	195	133.1	869.0	6.5
[CrOF_4_]^−^	200	248.5	970.5	3.9
[VOF_4_]^2–^	200	86.9	1209.0	13.9
[MoOCl_4_]^−^	219	203.7	1235.3	6.1
[CrOCl_4_]^−^	224	340.8	1468.6	4.3
[VOCl_4_]^2−^	224	154.4	1459.0	9.4
[Cu(NH_3_)_4_]^2+^	241	323.4	2691.2	8.3
[MoOBr_4_]^−^	263	477.9	3135.4	6.6
[CrOBr_4_]^−^	268	851.6	2877.7	3.4
[VOBr_4_]^2–^	268	502.5	3499.9	7.0
[Ni(mnt)_2_]^−^	565	4468.1	36,676.4	8.2

a Measured with Intel Xeon Gold 6342 processors.

## Conclusions

We have developed, implemented, and tested a state-interaction approach for computing *g*-matrices based on nonrelativistic TDDFT and TDA states. This method constructs a SOC-dressed effective Hamiltonian and expresses angular momentum and spin operators on the basis of the target Kramers multiplet. The methodology was validated through *g*-shift calculations on a diverse set of molecular systems, including light-atom molecules, heavy-element compounds, and transition-metal complexes. Comparisons with CPKS and experimental data highlight the strengths and limitations of our approach. While our primary aim is not to deliver benchmark-level *g*-shift values, these comparisons serve to validate our implementation and illustrate the practical advantages of the state-interaction methodology.

The key advantages of this method over conventional one-component DFT approaches (e.g., CPKS) are (i) the ability to analyze *g*-shifts in terms of nonrelativistic states, and (ii) the inclusion of second- and higher-order SOC effects via quasi-degenerate perturbation theory (QDPT), which are essential for certain Δ*g* components, such as the parallel shift in nitrogen halides and monohydrides. By decomposing *g*-shifts into individual state contributions, our approach provides insights into the convergence of the effective Hamiltonian and the underlying mechanisms driving the shifts. Additionally, analyzing *g*-parameter variations with SOC scaling allows us to identify dominant SOC contributions and distinguish shifts originating from angular momentum versus spin interactions.

For molecules without heavy elements, our method produces results comparable to those of CPKS, while its performance declines in systems containing elements such as Hg and Ir. The superiority of TDDFT/TDA over CPKS is evident in cases where second-order SOC contributions are critical, as seen in Δ*g*_∥_ for nitrogen halides and monohydrides. For 3d transition-metal complexes, both TDDFT/TDA and CPKS yield results that agree well with experimental values; however, both methods fail to reproduce *g*-shifts in the studied 4d complex, [MoOBr_4_]^−^. The accuracy of Δ*g* values in some metal complexes, such as [Ni­(mnt)_2_]^−^, is highly sensitive to the choice of the exchange–correlation functional, specifically the fraction of HFX, which modulates metal–ligand covalency and spin-density delocalization.

Discrepancies between CPKS and TDDFT/TDA *g*-shifts appear to stem at least in part from spin contamination in excited states contributing to the state-interaction treatment. This highlights the importance of spin purity in accurately modeling SOC effects within TDDFT-based approaches. While our analysis suggests a role for spin contamination in shaping the computed *g*-parameters, a comprehensive understanding of its impact will require further investigation.

Finally, it is important to acknowledge that our implementation inherits the advantages and limitations of both the underlying nonrelativistic approach (TDDFT/TDA) and the treatment of relativistic effects via the BP Hamiltonian. While BP mean-field SOCs provide a reasonable description for molecules without very heavy elements, they are insufficient for accurately capturing relativistic effects in heavier atoms, potentially explaining the discrepancies observed for molecules such as HgH and IrH_2_.

## Supplementary Material



## Data Availability

The data that supports the findings of this study are available within the article and its Supporting Information.

## Notes

Computational details, analysis of spin contamination in excited states and additional results for the studied molecules (PDF)
