# Supplementary material for: State-Interaction Approach for g‑Matrix Calculations in TDDFT: Ground-Excited State Couplings and beyond First-Order Spin–Orbit Effects
Source: J Chem Theory Comput. 2025 Jun 18;21(13):6528–44. doi: 10.1021/acs.jctc.5c00514 (PMC12243091; doi:10.1021/acs.jctc.5c00514)
Supplement: Supplementary file 1 [file ct5c00514_si_001.pdf]

Supporting Information: State-Interaction Approach for g-Matrix Calculations in TDDFT: Ground-Excited State Couplings and Beyond First-Order Spin-Orbit Effects

Antonio Cebreiro[†,‡] and David Casanova[*,†,¶]

†*Donostia International Physics Center (DIPC), 20018 Donostia, Euskadi, Spain*

‡*Polimero eta Material Aurreratuak: Fisika, Kimika eta Teknologia Saila, Kimika Fakultatea, Euskal Herriko Unibertsitatea (UPV/EHU), PK 1072, 20080 Donostia, Euskadi, Spain*

¶*IKERBASQUE, Basque Foundation for Science, 48009 Bilbao, Euskadi, Spain*

E-mail: david.casanova@dipc.org

Contents

1 Computational details **S3**

1.1 Python scripts for state-interaction calculations of g-matrix S3

1.2 Q-Chem input example: TDDFT S3

1.3 ORCA input example: CPKS S4

1.4 Molecular geometries S4

2 Spin contamination in TDDFT/TDA states **S9**

2.1 Spin contamination of contributing excited states using B3LYP functional . . S13

2.2 Spin contamination of contributing excited states using BLYP functional . . S15

3 Light atom molecules **S17**

3.1 Sum-over-state plots S17

3.2 Functionals comparison in the Δg of NO_2 and ${CO_2}^-$ S18

3.3 Dispersion correction with SCNL and NL S20

4 Different terms in the CPKS *g*-shift **S21**

5 Heavy atom molecules **S24**

6 Transition metal complexes **S25**

6.1 Individual excited state contributions S25

6.2 Spin density plots S29

6.3 Comparison of common gauge origin (CGO) and gauge including atomic orbitals (GIAOs) S30

6.4 Exchange-correlation functional dependence S30

7 Convergence analysis **S33**

8 g-shifts with BLYP functional **S34**

References **S35**

1 Computational details

1.1 Python scripts for state-interaction calculations of g-matrix

We implemented the calculations of g-shifts using the state-interaction approach in *Python* scripts. The scripts are available for download in *PyQChem*.[S1] The calculation is split into two steps: (i) parsing *Q-Chem* outputs and generating a *json* input file with the dictionaries shown below (ii) calculation of g-matrix parameters following the procedure by Tatchen *et. al.*[S2]

```
{
 "energy_dict":{
  <Excitation energies>
 },
 "soc_matrix_dict":{
  <Spin-orbit couplings>
 },
 "spin_dict":{
  <States spin>
 },
 "angmoment_dict":{
  <Orbital Angular Momentum>
 }
}
```

1.2 Q-Chem input example: TDDFT

```
$comment
 TD-DFT/TDA CALCULATION
$end

$molecule
 READ ni.molecule
$end

$rem
JOBTYPE            SP
EXCHANGE           B3LYP
CORRELATION        NONE
BASIS              GEN
PURECART     111
MAX_SCF_CYCLES     500
SCF_CONVERGENCE     8
THRESH             12
CIS_N_ROOTS        100
MAX_CIS_CYCLES     200
CALC_SOC           2
STS_ANGMOM         TRUE
RPA                FALSE !0: TDDFT/TDA, 1: both TDA and full
$end

$basis
!Specify ANO-RCC-TZVP basis
$end
```

1.3 ORCA input example: CPKS

```
!B3LYP ANO-RCC-TZP SOMF(1X) LARGEPRINT PrintMOs

%METHOD
 Functional B3LYP_G
 RI false
END

%SCF
 MaxIter 500
 Thresh 1e-12
 TolE 1e-8
END

%EPRNMR
 gtensor 1
 ori CenterOfNucCharge
 printlevel 4
END

* gzmtfile 0 3 ni.gzmt
```

1.4 Molecular geometries

Table S1: Bond lengths and angles of selected diatomic and triatomic molecules.

Molecule	X–Y (Å)	∠(Y–X–Y) (°)
H_2O^+	1.1056	109.62
NO_2	1.1944	134.23
CO_2^-	1.2535	133.88
NBr	1.808	—
NI	2.007	—
PdH	1.529	—
CdH	1.781	—
HgH	1.766	—
RhH_2	1.510	84.00
IrH_2	1.540	91.90

Cartesian geometries for transition atom complexes (in Å):

- $[CrOBr_4]^-$

```
Cr 0.000000 0.000000 0.000000

O 0.000000 0.000000 1.554000

Br 2.331661 0.000000 -0.577028

Br 0.000000 2.331661 -0.577028
```

Br -2.331661 0.000000 -0.577028

Br -0.000000 -2.331661 -0.577028

- $[CrOCl_4]^-$

Cr 0.000000 0.000000 0.000000

O 0.000000 0.000000 1.554000

Cl 2.171543 0.000000 -0.545454

Cl 0.000000 2.171543 -0.545454

Cl -2.171543 0.000000 -0.545454

Cl -0.000000 -2.171543 -0.545454

- $[CrOF_4]^-$

Cr 0.000000 0.000000 0.000000

O 0.000000 0.000000 1.562000

F 1.725593 0.000000 -0.472069

F 0.000000 1.725593 -0.472069

F -1.725593 0.000000 -0.472069

F -0.000000 -1.725593 -0.472069

- $[CuCl_4]^{2-}$

Cu 0.000000000 0.000000000 0.000000000

Cl 2.311187145 0.000193962 0.000000000

Cl 0.000000000 2.311051324 0.000000000

Cl -0.000300148 -2.311017743 -0.000002063

Cl -2.311184508 -0.000191324 -0.000001445

- $[Cu(NH_3)_4]^{2+}$

Cu 0.000000000 0.000000000 0.000000000

N 2.069792801 -0.000154321 0.000000000

H 2.505629535 0.929497774 0.002402263

```
H    2.449007235  -0.480559296  -0.826605894
H    2.449000743  -0.484842588   0.824073697
N    0.000226341  -2.069317025  -0.000084937
H   -0.480514452  -2.448524769  -0.826508323
H   -0.483911864  -2.449104555   0.824065649
H    0.930005537  -2.504881604   0.001655634
N   -2.069872326   0.000035799  -0.000084931
H   -2.505703662  -0.929614259   0.000861464
H   -2.448912402   0.483428698   0.824837149
H   -2.449124383   0.481833183  -0.825845134
N    0.000000000   2.069316351   0.000000000
H    0.480213314   2.448208910   0.826866142
H    0.484816003   2.449083940  -0.823753797
H   -0.929693551   2.505065105  -0.002401724
```

- $[MoOBr_4]^-$

```
Mo 0.000000 0.000000 0.000000
O 0.000000 0.000000 1.659000
Br 2.412075 0.000000 -0.641803
Br 0.000000 2.412075 -0.641803
Br -2.412075 0.000000 -0.641803
Br -0.000000 -2.412075 -0.641803
```

- $[MoOCl_4]^-$

```
Mo 0.000000 0.000000 0.000000
O 0.000000 0.000000 1.661000
Cl 2.259206 0.000000 -0.609581
Cl 0.000000 2.259206 -0.609581
Cl -2.259206 0.000000 -0.609581
Cl -0.000000 -2.259206 -0.609581
```

- $[MoOF_4]^-$

Mo 0.000000 0.000000 0.000000

O 0.000000 0.000000 1.670000

F 1.829279 0.000000 -0.573261

F 0.000000 1.829279 -0.573261

F -1.829279 0.000000 -0.573261

F -0.000000 -1.829279 -0.573261

- $[Ni(mnt)_2]^-$

Ni	-0.000319	0.043438	0.024183
S	1.535313	0.719301	-1.365674
S	1.495374	-0.590155	1.476136
S	-1.492799	0.663657	-1.438089
S	-1.540462	-0.632875	1.409174
C	4.197307	-0.534735	1.430447
C	4.236412	0.688318	-1.231282
N	5.209982	0.962972	-1.816932
N	5.150459	-0.801988	2.051872
C	3.015336	-0.217493	0.711251
C	3.033351	0.363881	-0.550964
C	-4.197138	0.547637	-1.421624
C	-4.238175	-0.657500	1.238051
N	-5.213283	-0.951754	1.809858
N	-5.156720	0.788327	-2.044142
C	-3.014384	0.266527	-0.689271
C	-3.036334	-0.308973	0.575648

- TiF_3

Ti	0.000000	0.000000	0.000000

```
F     1.756834    0.000000    0.000000
F    -0.878417    1.521463    0.000000
F    -0.878417   -1.521463   -0.000000
```

- $[VOBr_4]^{2-}$

```
V 0.000000 0.000000 0.000000
O 0.000000 0.000000 1.590000
Br 2.474931 0.000000 -0.557750
Br 0.000000 2.474931 -0.557750
Br -2.474931 0.000000 -0.557750
Br -0.000000 -2.474931 -0.557750
```

- $[VOCl_4]^{2-}$

```
V 0.000000 0.000000 0.000000
O 0.000000 0.000000 1.592000
Cl 2.319037 0.000000 -0.539657
Cl 0.000000 2.319037 -0.539657
Cl -2.319037 0.000000 -0.539657
Cl -0.000000 -2.319037 -0.539657
```

- $[VOF_4]^{2-}$

```
V 0.000000 0.000000 0.000000
O 0.000000 0.000000 1.615000
F 1.851307 0.000000 -0.523867
F 0.000000 1.851307 -0.523867
F -1.851307 0.000000 -0.523867
F -0.000000 -1.851307 -0.523867
```

2 Spin contamination in TDDFT/TDA states

Table S2: Comparison of spin contamination for TDDFT/TDA states computed using B3LYP and BLYP functionals. States included are those with the largest contribution to the g-matrix.

Molecule	B3LYP				BLYP			
	State	Calc. $\langle \hat{S}^2 \rangle$	Target $\langle \hat{S}^2 \rangle$	Error	State	Calc. $\langle \hat{S}^2 \rangle$	Target $\langle \hat{S}^2 \rangle$	Error
CdH	1	0.754	0.75	0.004	1	0.753	0.75	0.003
	2	0.759	0.75	0.009	2	0.76	0.75	0.01
	3	0.759	0.75	0.009	3	0.76	0.75	0.01
${CO_2}^-$	1	0.753	0.75	0.003	1	0.752	0.75	0.002
	2	0.756	0.75	0.006	2	0.753	0.75	0.003
	4	0.776	0.75	0.026	4	0.771	0.75	0.021
	13	0.787	0.75	0.037		–	–	–
		–	–	–	14	0.786	0.75	0.036
	15	1.358	0.75	0.608		–	–	–
$[CrOBr_4]^-$	1	0.808	0.75	0.058	1	0.769	0.75	0.019
	16	0.945	0.75	0.195		–	–	–
		–	–	–	19	0.874	0.75	0.124
	42	1.968	0.75	1.218		–	–	–
	43	1.968	0.75	1.218		–	–	–
		–	–	–	48	1.951	0.75	1.201
		–	–	–	49	1.951	0.75	1.201
	57	1.632	0.75	0.882		–	–	–
	58	1.632	0.75	0.882		–	–	–
	61	0.86	0.75	0.11		–	–	–
$[CrOCl_4]^-$	1	0.794	0.75	0.044	1	0.765	0.75	0.015
	2	0.825	0.75	0.075	2	0.787	0.75	0.037
	3	0.825	0.75	0.075	3	0.787	0.75	0.037
	67	0.823	0.75	0.073		–	–	–

Molecule	B3LYP				BLYP			
	State	Calc. $\langle \hat{S}^2 \rangle$	Target $\langle \hat{S}^2 \rangle$	Error	State	Calc. $\langle \hat{S}^2 \rangle$	Target $\langle \hat{S}^2 \rangle$	Error
		–	–	–	70	0.812	0.75	0.062
$[CrOF_4]^-$	1	0.776	0.75	0.026	1	0.761	0.75	0.011
	2	0.779	0.75	0.029	2	0.766	0.75	0.016
	3	0.779	0.75	0.029	3	0.766	0.75	0.016
	4	0.787	0.75	0.037		–	–	–
		–	–	–	5	0.768	0.75	0.018
$[CuCl_4]^{2-}$	1	0.753	0.75	0.003	1	0.751	0.75	0.001
	4	0.754	0.75	0.004		–	–	–
	11	0.758	0.75	0.008	11	0.752	0.75	0.002
	12	0.758	0.75	0.008	12	0.752	0.75	0.002
		–	–	–	13	0.752	0.75	0.002
$[Cu(NH_3)_4]^{2+}$	1	0.753	0.75	0.003	1	0.751	0.75	0.001
	3	0.753	0.75	0.003	3	0.751	0.75	0.001
	4	0.753	0.75	0.003	4	0.751	0.75	0.001
	5	0.753	0.75	0.003	5	0.752	0.75	0.002
HgH	1	0.75	0.75	0.0		–	–	–
	2	0.751	0.75	0.001		–	–	–
	3	0.751	0.75	0.001		–	–	–
IrH_2	1	0.759	0.75	0.009		–	–	–
	2	0.755	0.75	0.005		–	–	–
$[MoOBr_4]^-$	1	0.756	0.75	0.006	1	0.754	0.75	0.004
	5	0.772	0.75	0.022		–	–	–
		–	–	–	8	0.763	0.75	0.013
		–	–	–	13	0.756	0.75	0.006
		–	–	–	14	0.756	0.75	0.006
		–	–	–	59	0.77	0.75	0.02
	63	1.212	0.75	0.462		–	–	–

Molecule	B3LYP				BLYP			
	State	Calc. $\langle \hat{S}^2 \rangle$	Target $\langle \hat{S}^2 \rangle$	Error	State	Calc. $\langle \hat{S}^2 \rangle$	Target $\langle \hat{S}^2 \rangle$	Error
	64	1.212	0.75	0.462		–	–	–
$[MoOCl_4]^-$	1	0.755	0.75	0.005	1	0.753	0.75	0.003
	2	0.76	0.75	0.01	2	0.758	0.75	0.008
	3	0.76	0.75	0.01	3	0.758	0.75	0.008
	55	0.774	0.75	0.024		–	–	–
		–	–	–	67	0.771	0.75	0.021
$[MoOF_4]^-$	1	0.754	0.75	0.004	1	0.753	0.75	0.003
	2	0.755	0.75	0.005	2	0.754	0.75	0.004
	3	0.755	0.75	0.005	3	0.754	0.75	0.004
		–	–	–	6	0.755	0.75	0.005
	88	0.791	0.75	0.041		–	–	–
NBr	1	2.01	2.0	0.01	1	2.007	2.0	0.007
	2	2.168	2.0	0.168		–	–	–
	3	2.168	2.0	0.168		–	–	–
	7	2.039	2.0	0.039	7	2.036	2.0	0.036
	8	2.039	2.0	0.039	8	2.036	2.0	0.036
	10	3.647	6.0	-2.353	10	3.834	6.0	-2.166
	11	3.647	6.0	-2.353	11	3.834	6.0	-2.166
NI	1	2.013	2.0	0.013	1	2.008	2.0	0.008
	2	2.296	2.0	0.296		–	–	–
	3	2.296	2.0	0.296		–	–	–
		–	–	–	4	2.143	2.0	0.143
		–	–	–	5	2.143	2.0	0.143
		–	–	–	7	2.069	2.0	0.069
		–	–	–	8	2.069	2.0	0.069
	10	3.473	2.0	1.473		–	–	–
	11	3.473	2.0	1.473		–	–	–
$[Ni(mnt)_2]^-$	1	0.76	0.75	0.01	1	0.753	0.75	0.003

Molecule	B3LYP				BLYP			
	State	Calc. $\langle \hat{S}^2 \rangle$	Target $\langle \hat{S}^2 \rangle$	Error	State	Calc. $\langle \hat{S}^2 \rangle$	Target $\langle \hat{S}^2 \rangle$	Error
	2	0.772	0.75	0.022	2	0.755	0.75	0.005
	3	0.764	0.75	0.014		–	–	–
		–	–	–	4	0.756	0.75	0.006
		–	–	–	7	0.851	0.75	0.101
	18	1.474	0.75	0.724		–	–	–
	23	0.908	0.75	0.158		–	–	–
		–	–	–	43	0.929	0.75	0.179
NO_2	1	0.754	0.75	0.004	1	0.752	0.75	0.002
	2	0.755	0.75	0.005	2	0.752	0.75	0.002
	7	1.117	0.75	0.367	7	0.887	0.75	0.137
	8	0.759	0.75	0.009	8	0.755	0.75	0.005
PdH	1	0.752	0.75	0.002		–	–	–
	4	0.756	0.75	0.006		–	–	–
	5	0.756	0.75	0.006		–	–	–
RhH_2	1	0.752	0.75	0.002	1	0.751	0.75	0.001
	2	0.753	0.75	0.003	2	0.752	0.75	0.002
	3	0.753	0.75	0.003	3	0.751	0.75	0.001
SeO	1	2.009	2.0	0.009	1	2.007	2.0	0.007
	5	2.022	2.0	0.022	5	2.012	2.0	0.012
	6	2.022	2.0	0.022	6	2.012	2.0	0.012
	7	2.023	2.0	0.023	7	2.034	2.0	0.034
	8	2.023	2.0	0.023	8	2.034	2.0	0.034
TiF_3	1	0.752	0.75	0.002	1	0.752	0.75	0.002
	2	0.752	0.75	0.002	2	0.752	0.75	0.002
	3	0.752	0.75	0.002	3	0.752	0.75	0.002
	96	1.492	0.75	0.742		–	–	–
		–	–	–	98	1.403	0.75	0.653
$[VOBr_4]^{2-}$	1	0.767	0.75	0.017	1	0.759	0.75	0.009

Molecule	B3LYP				BLYP			
	State	Calc. $\langle \hat{S}^2 \rangle$	Target $\langle \hat{S}^2 \rangle$	Error	State	Calc. $\langle \hat{S}^2 \rangle$	Target $\langle \hat{S}^2 \rangle$	Error
		–	–	–	2	0.768	0.75	0.018
	3	0.771	0.75	0.021	3	0.768	0.75	0.018
	4	0.771	0.75	0.021		–	–	–
	58	0.805	0.75	0.055		–	–	–
		–	–	–	84	1.455	0.75	0.705
		–	–	–	86	1.76	0.75	1.01
$[VOCl_4]^{2-}$	1	0.764	0.75	0.014	1	0.758	0.75	0.008
	2	0.767	0.75	0.017	2	0.764	0.75	0.014
	3	0.767	0.75	0.017	3	0.764	0.75	0.014
	4	0.769	0.75	0.019	4	0.764	0.75	0.014
$[VOF_4]^{2-}$	1	0.759	0.75	0.009	1	0.756	0.75	0.006
	2	0.76	0.75	0.01	2	0.758	0.75	0.008
	3	0.76	0.75	0.01	3	0.758	0.75	0.008
	4	0.761	0.75	0.011		–	–	–
		–	–	–	5	0.759	0.75	0.009

2.1 Spin contamination of contributing excited states using B3LYP functional

This section offers the plots with the spin contamination in proportion to the g-shift of each excited state, calculated with equation 1.

$$\sigma_k = \left(\langle S^2 \rangle_{calc} - \langle S^2 \rangle_{ideal}\right) \cdot \Delta g_{kk}, \text{ for } k = x, y, z \quad (1)$$

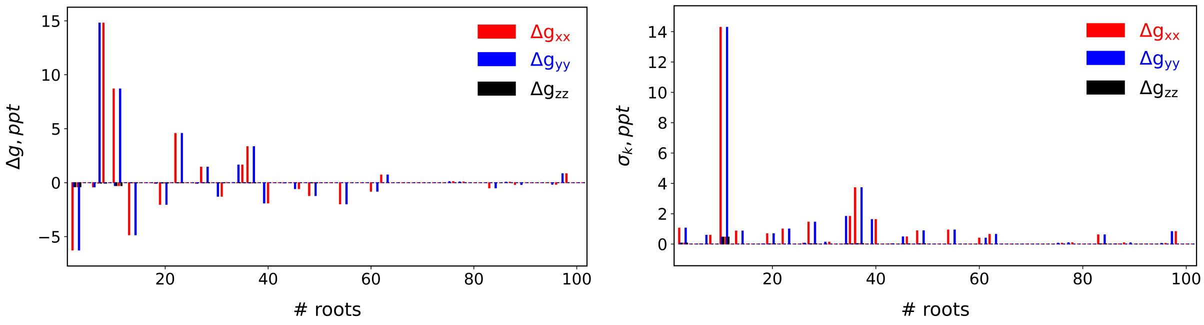

Figure S1: On the left, Δg in two-state SOC-dressed Hamiltonians, the ground and a single excited state (# roots in the x-axis) for NBr. On the right, $\langle S^2 \rangle$ contamination multiplied by the computed Δg. $\Delta g_z \equiv \Delta g_{\|}$; $\Delta g_{x,y} \equiv \Delta g_{\perp}$.

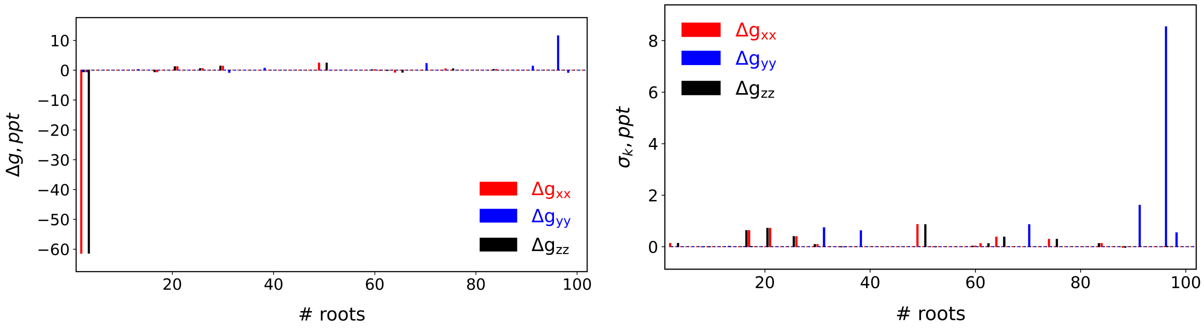

Figure S2: On the left, Δg in two-state SOC-dressed Hamiltonians, the ground and a single excited state (# roots in the x-axis) for TiF_3. On the right, $\langle S^2 \rangle$ contamination multiplied by the computed Δg. $\Delta g_y \equiv \Delta g_{\|}$; $\Delta g_{x,z} \equiv \Delta g_{\perp}$.

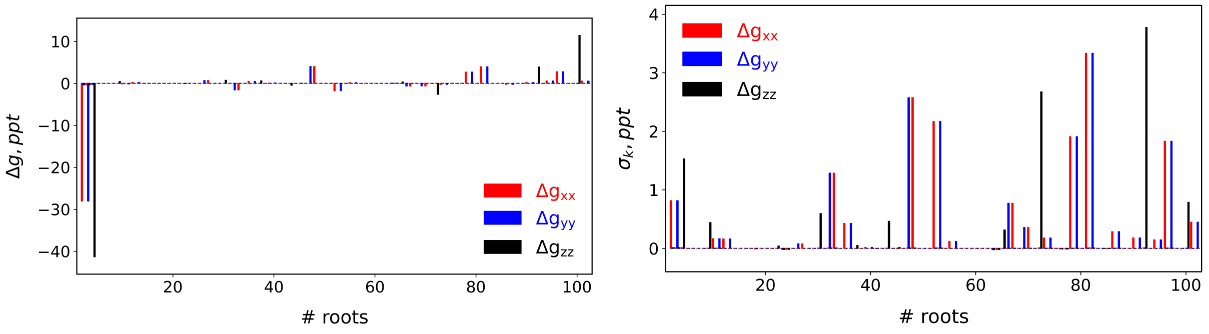

Figure S3: On the left, Δg in two-state SOC-dressed Hamiltonians, the ground and a single excited state (# roots in the x-axis) for $[CrOF_4]^-$. On the right, $\langle S^2 \rangle$ contamination multiplied by the computed Δg. $\Delta g_z \equiv \Delta g_{\|}$; $\Delta g_{x,y} \equiv \Delta g_{\perp}$.

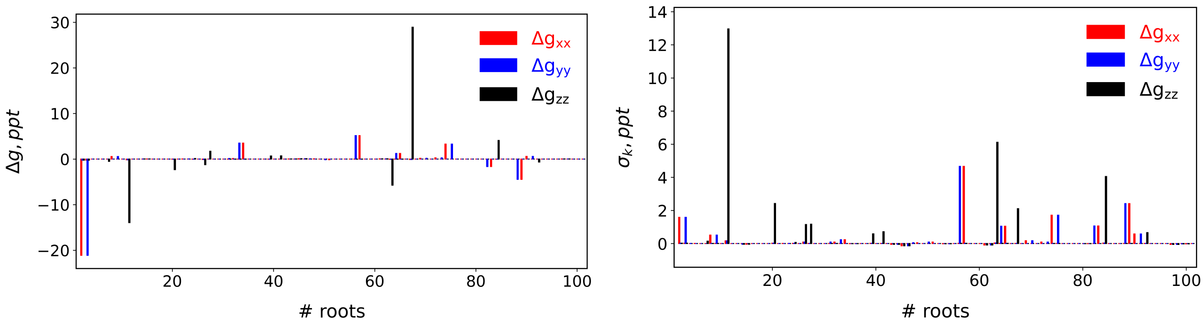

Figure S4: On the left, Δg in two-state SOC-dressed Hamiltonians, the ground and a single excited state (# roots in the x-axis) for $[CrOCl_4]^-$. On the right, $\langle S^2 \rangle$ contamination multiplied by the computed Δg. $\Delta g_z \equiv \Delta g_{\parallel}$; $\Delta g_{x,y} \equiv \Delta g_{\perp}$.

2.2 Spin contamination of contributing excited states using BLYP functional

This section offers the plots with the spin contamination in proportion to the g-shift of each excited state, calculated with equation 1.

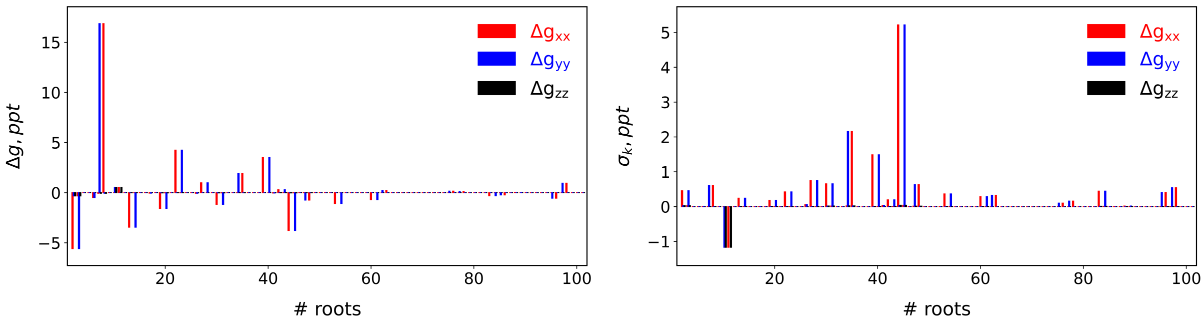

Figure S5: On the left, Δg in two-state SOC-dressed Hamiltonians, the ground and a single excited state (# roots in the x-axis) for NBr. On the right, $\langle S^2 \rangle$ contamination multiplied by the computed Δg. $\Delta g_z \equiv \Delta g_{\parallel}$; $\Delta g_{x,y} \equiv \Delta g_{\perp}$.

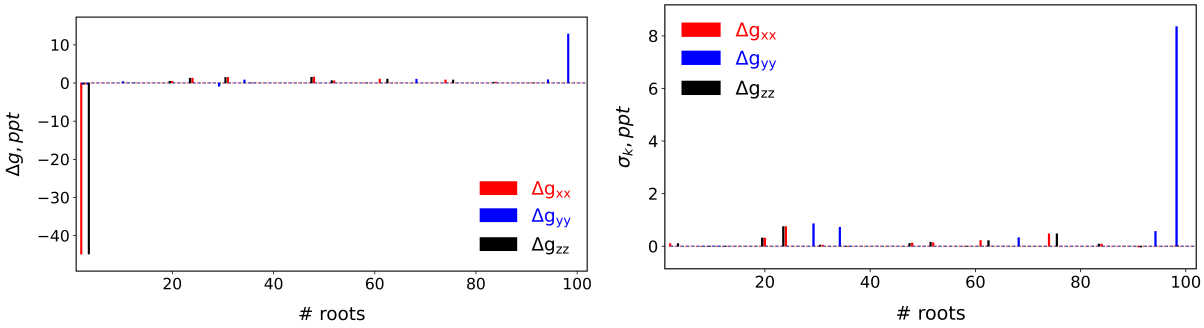

Figure S6: On the left, Δg in two-state SOC-dressed Hamiltonians, the ground and a single excited state (# roots in the x-axis) for TiF_3. On the right, $\langle S^2 \rangle$ contamination multiplied by the computed Δg. $\Delta g_y \equiv \Delta g_{\|}$; $\Delta g_{x,z} \equiv \Delta g_{\perp}$.

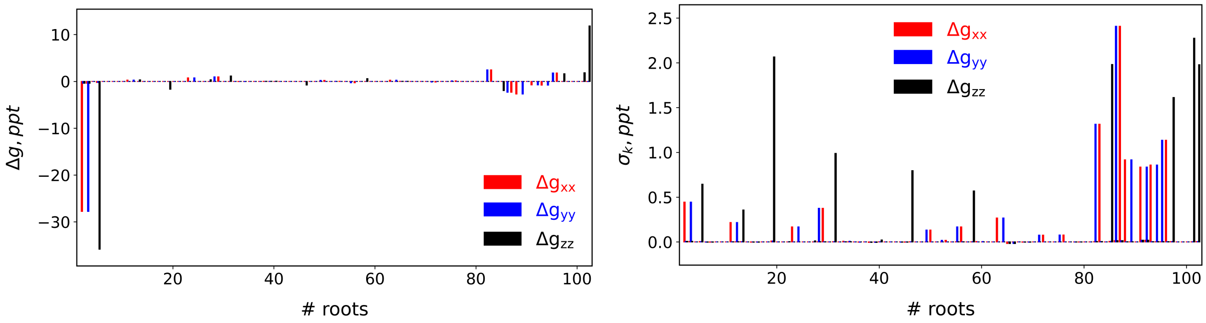

Figure S7: On the left, Δg in two-state SOC-dressed Hamiltonians, the ground and a single excited state (# roots in the x-axis) for $[CrOF_4]^-$. On the right, $\langle S^2 \rangle$ contamination multiplied by the computed Δg. $\Delta g_z \equiv \Delta g_{\|}$; $\Delta g_{x,y} \equiv \Delta g_{\perp}$.

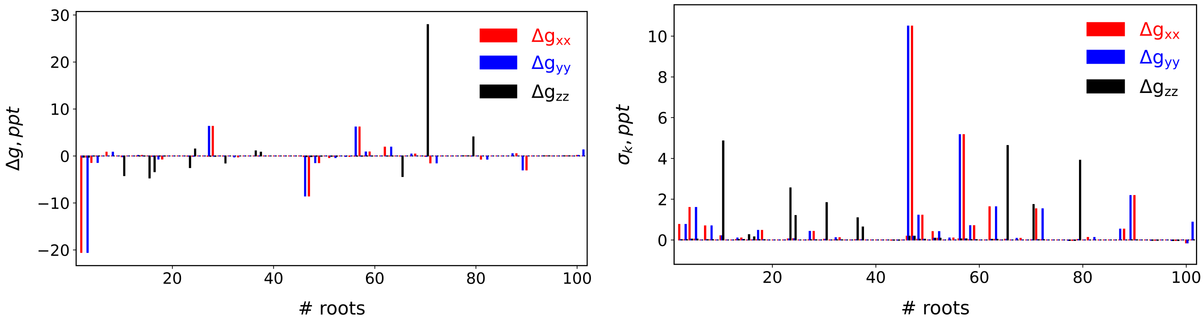

Figure S8: On the left, Δg in two-state SOC-dressed Hamiltonians, the ground and a single excited state (# roots in the x-axis) for $[CrOCl_4]^-$. On the right, $\langle S^2 \rangle$ contamination multiplied by the computed Δg. $\Delta g_z \equiv \Delta g_{\|}$; $\Delta g_{x,y} \equiv \Delta g_{\perp}$.

3 Light atom molecules

3.1 Sum-over-state plots

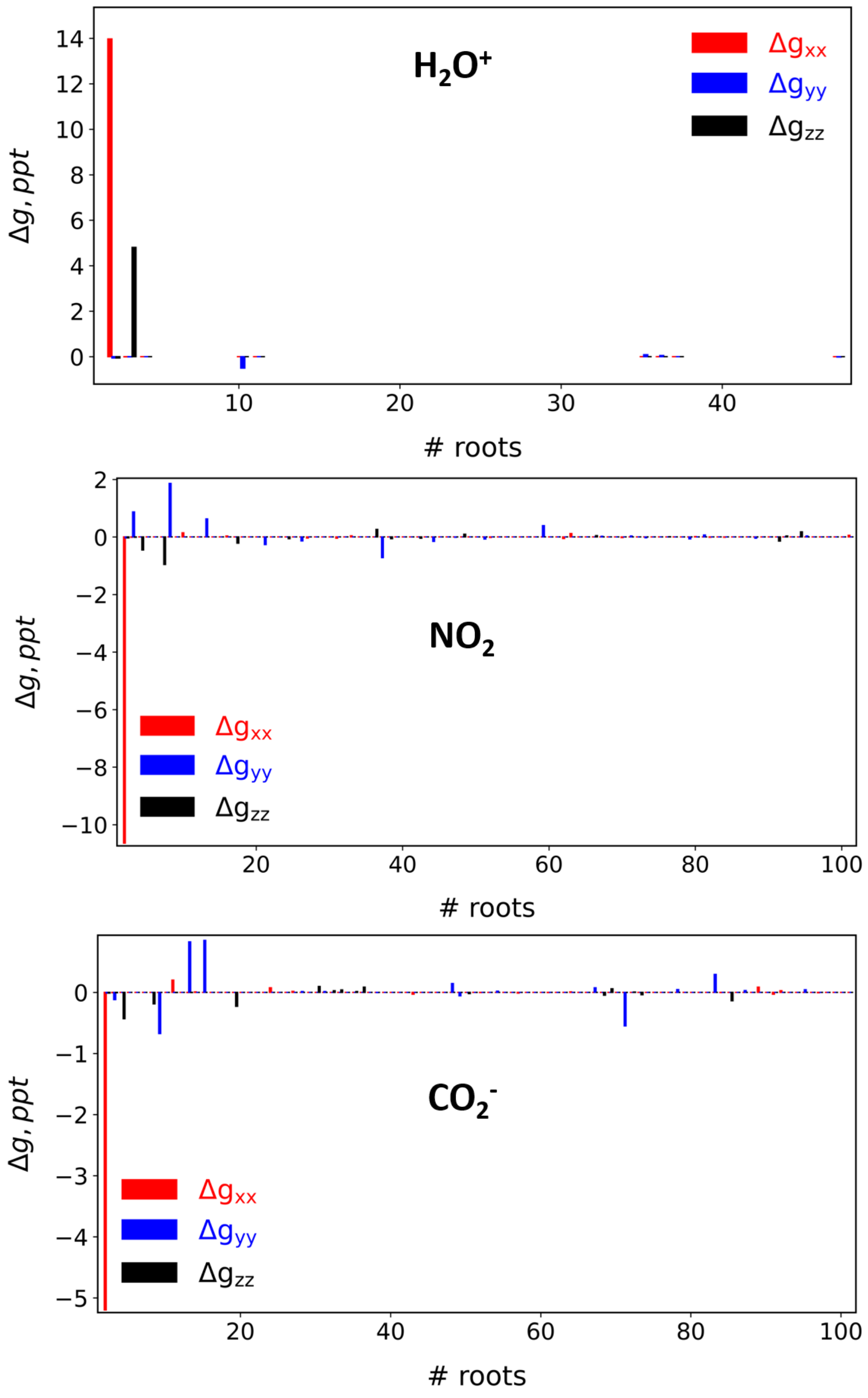

Figure S9: Δg in two-state SOC-dressed Hamiltonians, the ground and a single excited state (# roots in the x-axis) for H_2O^+, NO_2 and $CO_2{}^-$.

3.2 Functionals comparison in the Δg of NO_2 and CO_2^-

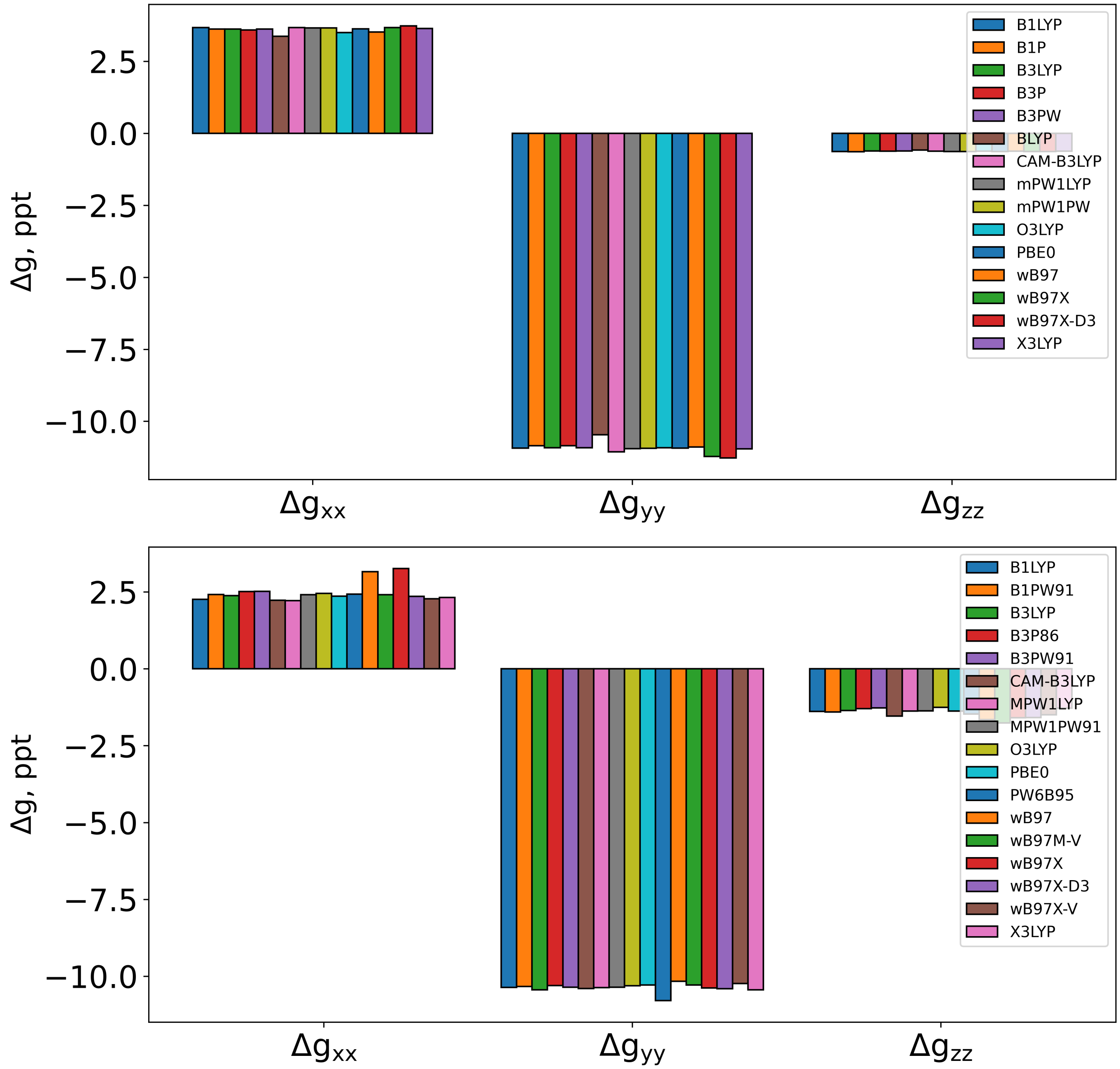

Figure S10: Results comparison for different functionals using CPKS (above) and TDDFT/TDA (below) in NO_2.

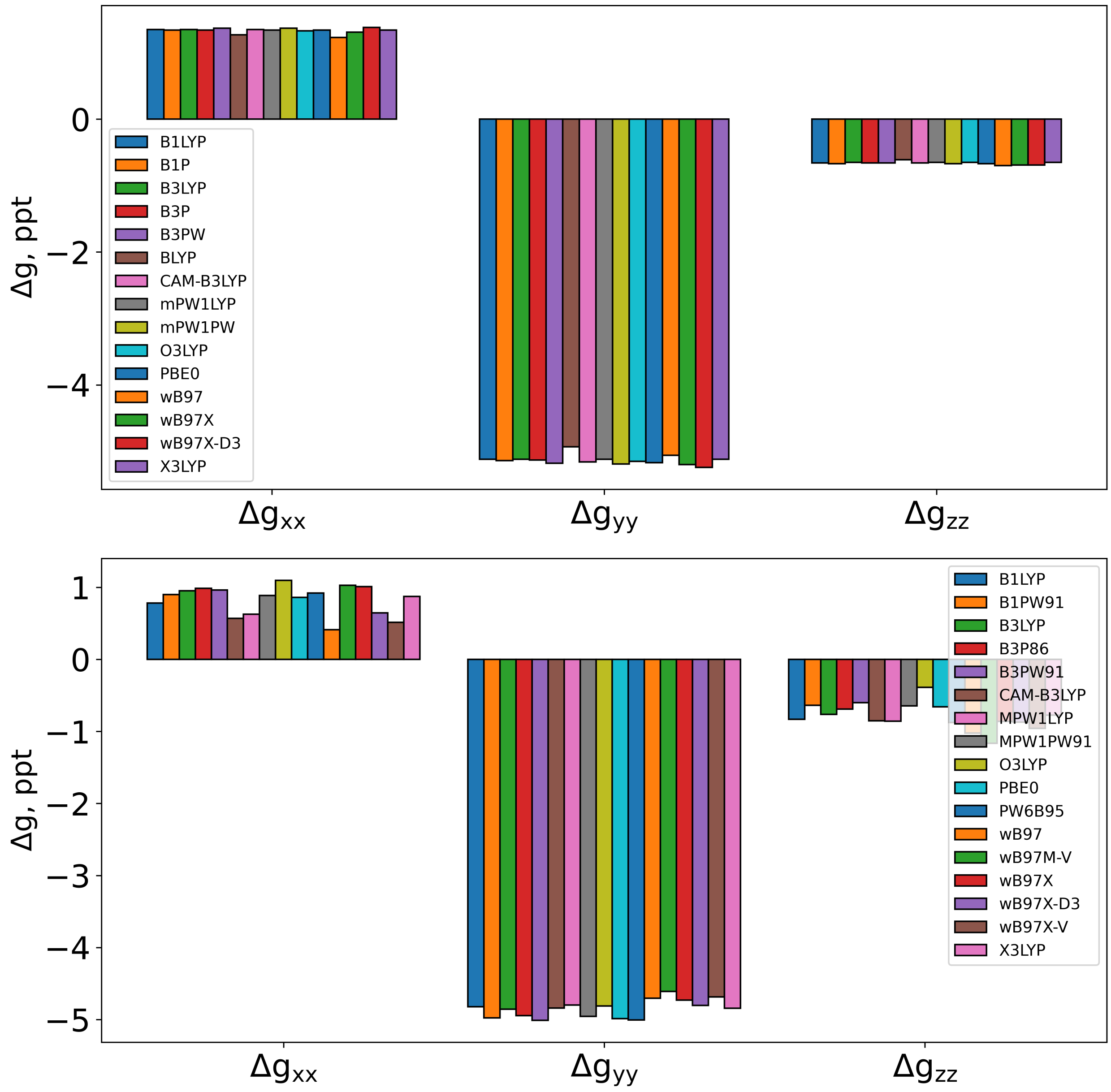

Figure S11: Results comparison for different functionals using CPKS (above) and TDDFT/TDA (below) in $CO_2{}^-$.

3.3 Dispersion correction with SCNL and NL

There are two different ways of computing the non-local VV10 kernel in ωB97X-V and ωB97M-V functionals: fully self-consistently or as an additive correction.[S3,S4] The two strategies seem not to have a significant effect on the energetic properties.[S4] No differences are observed in KS energy; however, as shown in the table S3, there is a significant difference in the main values of Δg.

Table S3: Calculated Total KS energy and $\Delta g_{\perp}$ values (in ppt) for small molecules with CP-KS using ωB97X-V and ωB97M-V functionals computed self-consistently (SCNL) and as additive correction (NL).

molecule	functional	SCNL				NL			
		E / E_h	Δg_{xx}	Δg_{yy}	Δg_{zz}	E / E_h	Δg_{xx}	Δg_{yy}	Δg_{zz}
H_2O^+	ωB97M	-75.959066	-0.19	12.97	4.25	-75.959065	-0.18	11.38	5.28
H_2O^+	ωB97X	-75.959754	-0.20	13.66	4.27	-75.959753	-0.19	12.70	5.42
NO_2	ωB97M	-205.112425	3.72	**-11.13**	-0.60	-205.112422	4.65	**-17.12**	-0.68
NO_2	ωB97X	-205.095078	3.68	**-10.96**	-0.60	-205.095074	4.55	**-15.90**	-0.68
CO_2^-	ωB97M	-188.589751	1.37	**-5.21**	-0.66	-188.589746	1.79	**-12.51**	-0.63
CO_2^-	ωB97X	-188.573829	1.37	**-5.06**	-0.65	-188.573824	1.75	**-8.86**	-0.62

4 Different terms in the CPKS g-shift

Table S4: Different Δg terms (in ppt) computed with CPKS/B3LYP: relativistic mass correction (RMC), gauge correction (GC, also known as diamagnetic spin-orbit term) and paramagnetic spin-orbit terms, along with their ratios with respect to total Δg (in %).

molecule		RMC	Ratio	GC	Ratio	PSO	Ratio	Δg
CdH	Δg_{xx}	-0.131	0.149	0.424	-0.483	-88.131	100.334	-87.837
	Δg_{yy}	-0.131	0.149	0.424	-0.483	-88.131	100.334	-87.837
	Δg_{zz}	-0.131	-90.764	0.275	190.764	0.0	0.0	0.144
CO_2^-	Δg_{xx}	-0.229	4.464	0.13	-2.545	-5.022	98.08	-5.121
	Δg_{yy}	-0.229	35.365	0.074	-11.525	-0.492	76.16	-0.646
	Δg_{zz}	-0.229	-16.865	0.14	10.299	1.445	106.566	1.355
$[CrOBr_4]^-$	Δg_{xx}	-0.725	2.251	0.196	-0.61	-31.673	98.358	-32.202
	Δg_{yy}	-0.725	2.251	0.196	-0.61	-31.673	98.358	-32.202
	Δg_{zz}	-0.725	-0.601	0.334	0.277	120.907	100.325	120.516
$[CrOCl_4]^-$	Δg_{xx}	-0.718	2.742	0.207	-0.791	-25.659	98.05	-26.169
	Δg_{yy}	-0.718	2.742	0.207	-0.791	-25.659	98.05	-26.169
	Δg_{zz}	-0.718	-4.052	0.341	1.926	18.083	102.125	17.707
$[CrOF_4]^-$	Δg_{xx}	-0.46	0.179	0.245	-0.095	-256.578	99.916	-256.793
	Δg_{yy}	-0.46	0.179	0.245	-0.095	-256.578	99.916	-256.793
	Δg_{zz}	-0.46	-1.541	0.367	1.23	29.916	100.312	29.823
$[CuCl_4]^{2-}$	Δg_{xx}	-0.924	-2.311	0.369	0.923	40.527	101.387	39.973
	Δg_{yy}	-0.924	-2.31	0.369	0.923	40.532	101.387	39.978
	Δg_{zz}	-0.924	-0.652	0.543	0.384	142.004	100.269	141.624
$[Cu(NH_3)_4]^+$	Δg_{xx}	-0.998	-2.481	0.342	0.849	40.902	101.631	40.246
	Δg_{yy}	-0.998	-2.479	0.342	0.848	40.932	101.63	40.275
	Δg_{zz}	-0.998	-0.68	0.505	0.344	147.311	100.336	146.817
H_2O^+	Δg_{xx}	-0.318	167.686	0.072	-38.06	0.056	-29.678	-0.19
	Δg_{yy}	-0.318	-7.638	0.148	3.563	4.334	104.075	4.165
	Δg_{zz}	-0.318	-2.535	0.149	1.19	12.719	101.345	12.551

molecule		RMC	Ratio	GC	Ratio	PSO	Ratio	Δg
HgH	Δg_{xx}	-0.057	-28.444	0.259	128.494	-0.0	-0.0	0.202
	Δg_{yy}	-0.057	-0.017	0.382	0.112	339.901	99.905	340.226
	Δg_{zz}	-0.057	-0.017	0.382	0.112	339.902	99.905	340.227
IrH_2	Δg_{xx}	-0.314	0.095	0.562	-0.169	-332.474	100.075	-332.226
	Δg_{yy}	-0.314	-118.484	0.441	166.352	0.138	52.169	0.265
	Δg_{zz}	-0.314	-0.009	0.812	0.022	-7624.148	-210.669	3619.012
$[MoOBr_4]^-$	Δg_{xx}	-0.229	-4.673	0.602	12.263	4.539	92.409	4.911
	Δg_{yy}	-0.229	-4.673	0.602	12.263	4.539	92.409	4.911
	Δg_{zz}	-0.229	-0.178	0.914	0.711	127.908	99.467	128.593
$[MoOCl_4]^-$	Δg_{xx}	-0.237	24.183	0.556	-56.791	-1.298	132.598	-0.979
	Δg_{yy}	-0.237	24.183	0.556	-56.791	-1.298	132.598	-0.979
	Δg_{zz}	-0.237	-0.818	0.859	2.968	28.317	97.85	28.939
$[MoOF_4]^-$	Δg_{xx}	-0.29	10.469	0.569	-20.53	-3.05	110.057	-2.771
	Δg_{yy}	-0.29	10.469	0.569	-20.53	-3.05	110.057	-2.771
	Δg_{zz}	-0.29	-4.068	0.873	12.244	6.548	91.824	7.131
NBr	Δg_{xx}	-0.243	371.669	0.177	-271.669	0.0	-0.0	-0.065
	Δg_{yy}	-0.243	-1.07	0.262	1.155	22.672	99.915	22.692
	Δg_{zz}	-0.243	-1.07	0.262	1.155	22.672	99.915	22.692
NI	Δg_{xx}	-0.239	-2279.048	0.25	2379.048	0.0	0.0	0.01
	Δg_{yy}	-0.239	-0.437	0.46	0.839	54.535	99.597	54.756
	Δg_{zz}	-0.239	-0.437	0.46	0.839	54.535	99.597	54.756
$[Ni(mnt)_2]^-$	Δg_{xx}	-0.561	10.209	0.335	-6.097	-5.265	95.888	-5.491
	Δg_{yy}	-0.561	-0.896	0.378	0.605	62.719	100.291	62.537
	Δg_{zz}	-0.561	-0.542	0.402	0.389	103.532	100.154	103.373
NO_2	Δg_{xx}	-0.326	2.982	0.166	-1.515	-10.78	98.532	-10.941
	Δg_{yy}	-0.326	53.282	0.105	-17.129	-0.391	63.863	-0.612
	Δg_{zz}	-0.326	-8.993	0.192	5.294	3.762	103.699	3.628
PdH	Δg_{xx}	-0.359	-741.031	0.408	841.031	-0.0	-0.0	0.049
	Δg_{yy}	-0.359	-0.257	0.648	0.463	139.499	99.794	139.787

molecule		RMC	Ratio	GC	Ratio	PSO	Ratio	Δg
	Δg_{zz}	-0.359	-0.257	0.648	0.463	139.499	99.794	139.787
RhH_2	Δg_{xx}	-0.543	220.732	0.544	-221.057	-0.247	100.366	-0.246
	Δg_{yy}	-0.543	-0.095	0.838	0.147	568.394	99.948	568.689
	Δg_{zz}	-0.543	-0.094	0.872	0.151	576.473	99.943	576.803
TiF_3	Δg_{xx}	-0.353	0.767	0.179	-0.389	-45.897	99.622	-46.072
	Δg_{yy}	-0.353	0.767	0.179	-0.389	-45.896	99.622	-46.071
	Δg_{zz}	-0.353	28.054	0.095	-7.514	-1.0	79.46	-1.259
$[VOBr_4]^{2-}$	Δg_{xx}	-0.549	2.501	0.173	-0.791	-21.557	98.29	-21.932
	Δg_{yy}	-0.549	2.501	0.173	-0.791	-21.557	98.29	-21.932
	Δg_{zz}	-0.549	-0.97	0.289	0.512	56.797	100.459	56.538
$[VOCl_4]^{2-}$	Δg_{xx}	-0.556	2.496	0.279	-1.252	-21.995	98.756	-22.272
	Δg_{yy}	-0.556	2.513	0.169	-0.762	-21.737	98.249	-22.125
	Δg_{zz}	-0.556	2.513	0.169	-0.762	-21.737	98.249	-22.125
$[VOF_4]^{2-}$	Δg_{xx}	-0.596	1.442	0.28	-0.677	-41.036	99.235	-41.353
	Δg_{yy}	-0.596	2.14	0.177	-0.636	-27.448	98.496	-27.868
	Δg_{zz}	-0.596	2.14	0.177	-0.636	-27.448	98.496	-27.868

5 Heavy atom molecules

In SeO, TDA $\Delta g_\perp$ mainly emerges from the interaction of the non-relativistic ground state with two doubly degenerated states with ΔE = 4.58 eV and SOCC = 714 cm^{-1}, and ΔE = 4.93 eV and SOCC = 658 cm^{-1}, respectively (states 5-6 and 7-8 in Figure S12). These values are in rather good agreement with recent results obtained with a RASCI wavefunction, showing $\Delta g_\perp$ = 15.1 ppt, with ΔE = 4.76 eV and SOCC = 743 cm^{-1}.[S5]

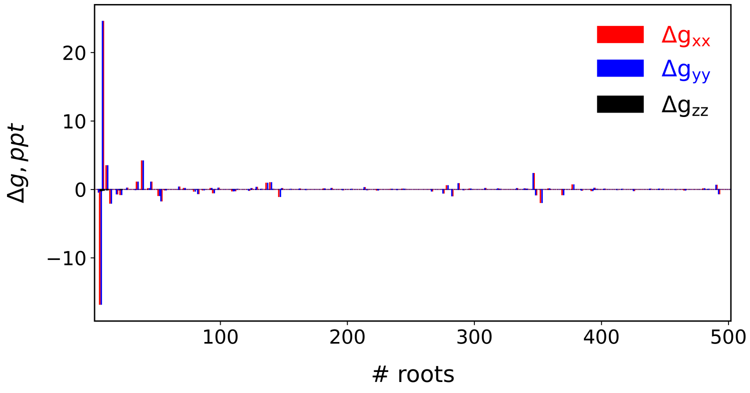

Figure S12: Δg in two-state SOC-dressed Hamiltonians, the ground and a single excited state (# roots in the x-axis) for SeO.

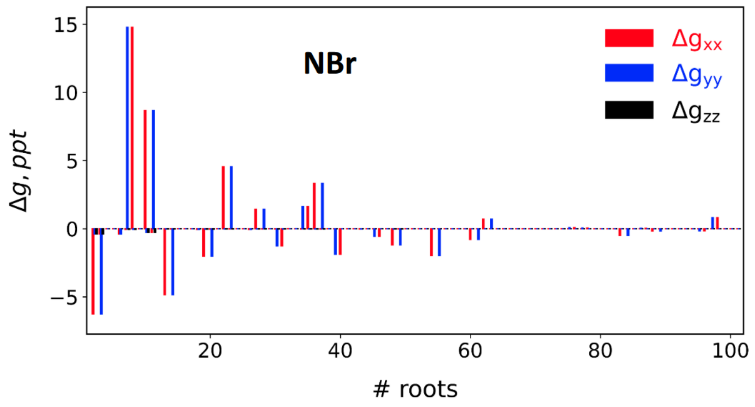

Figure S13: Δg in two-state SOC-dressed Hamiltonians, the ground and a single excited state (# roots in the x-axis) for NBr.

Table S5: Fitted parameters for the dependence of the perpendicular and parallel g-shifts on the SOC scaling factor in RhH_2 and IrH_2 compounds. The g-shift is modeled as $\Delta g = c_3\lambda^3 + c_2\lambda^2 + c_1\lambda + c_0$, where λ is the SOC scaling parameter, and Δg and the coefficients c_i $(i = 0, 3)$ are given in ppt. For all molecules, the fit achieves an accuracy of $R^2 = 1.0$. Z_{max} indicates the atomic number of the heaviest element.

molecule	Z_{max}	c_3	c_2	c_1	c_0
			Δg_{xx} component		
RhH_2	45	34.90	-153.05	-1.75	0.01
IrH_2	77	-679.57	2250.85	-2655.23	-3.08
			Δg_{yy} component		
RhH_2	45	1.60	-130.17	634.77	-0.17
IrH_2	77	399.18	-266.55	-1538.37	28.59
			Δg_{zz} component		
RhH_2	45	1.68	-134.58	626.34	-0.18
IrH_2	77	280.37	-87.66	-1061.12	24.70

6 Transition metal complexes

6.1 Individual excited state contributions

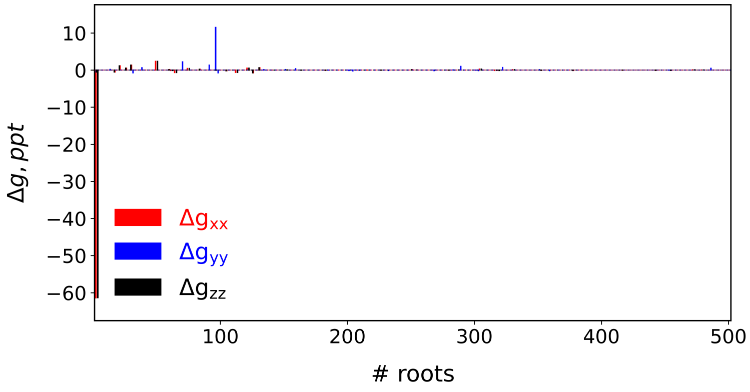

Figure S14: Δg in two-state SOC-dressed Hamiltonians, the ground and a single excited state (# roots in the x-axis) for TiF_3.

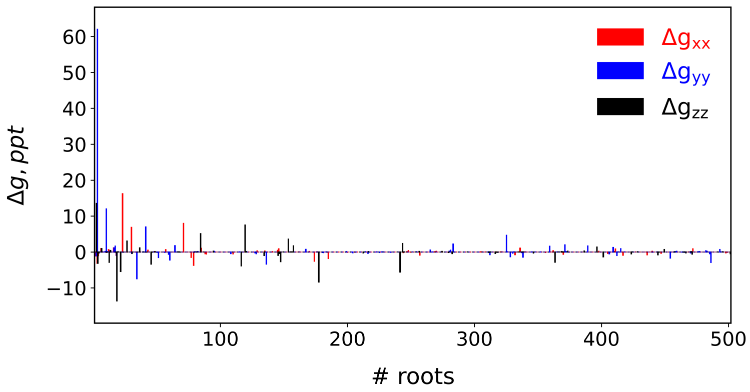

Figure S15: Δg in two-state SOC-dressed Hamiltonians, the ground and a single excited state (# roots in the x-axis) for $[Ni(mnt)_2]^-$.

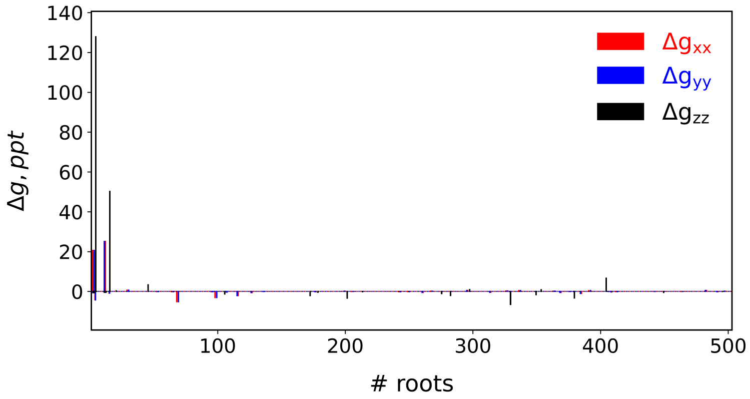

Figure S16: Δg in two-state SOC-dressed Hamiltonians, the ground and a single excited state (# roots in the x-axis) for $[CuCl_4]^{2-}$.

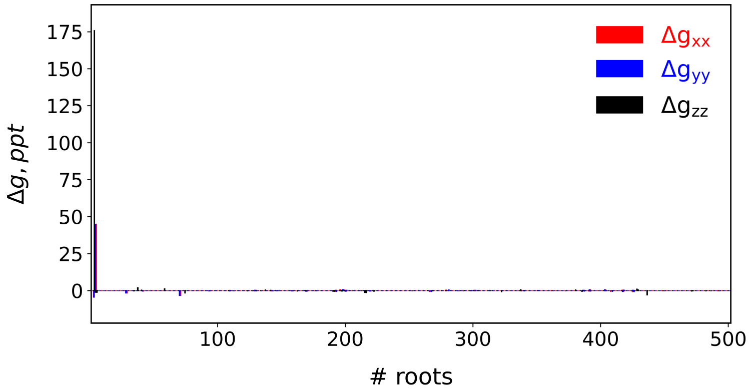

Figure S17: Δg in two-state SOC-dressed Hamiltonians, the ground and a single excited state (# roots in the x-axis) for $[Cu(NH_3)_4]^{2+}$.

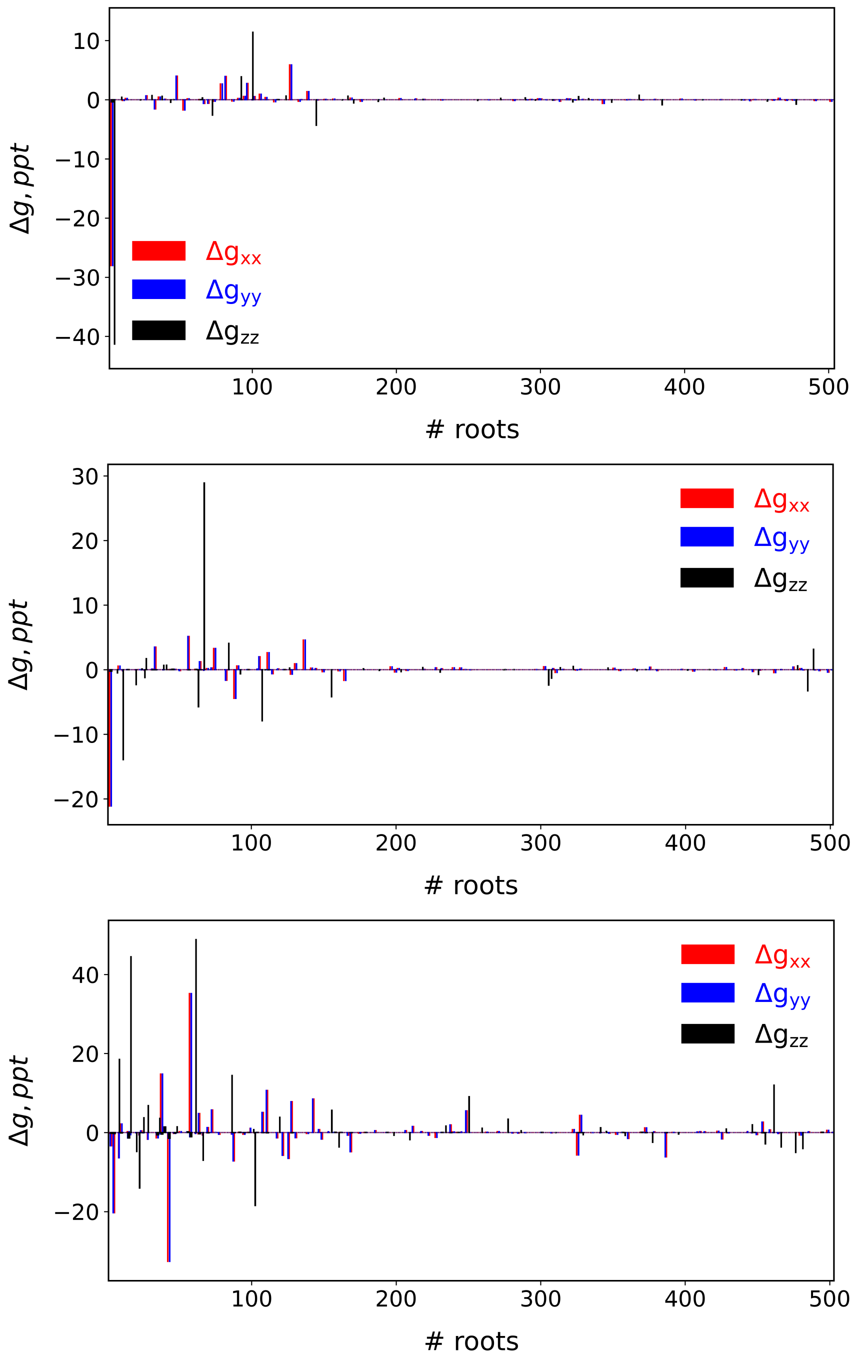

Figure S18: Δg components in two-state SOC-dressed Hamiltonians, the ground and a single excited state (# roots in the x-axis) for $CrOX_4^-$. From upper to lower, X=F,Cl,Br

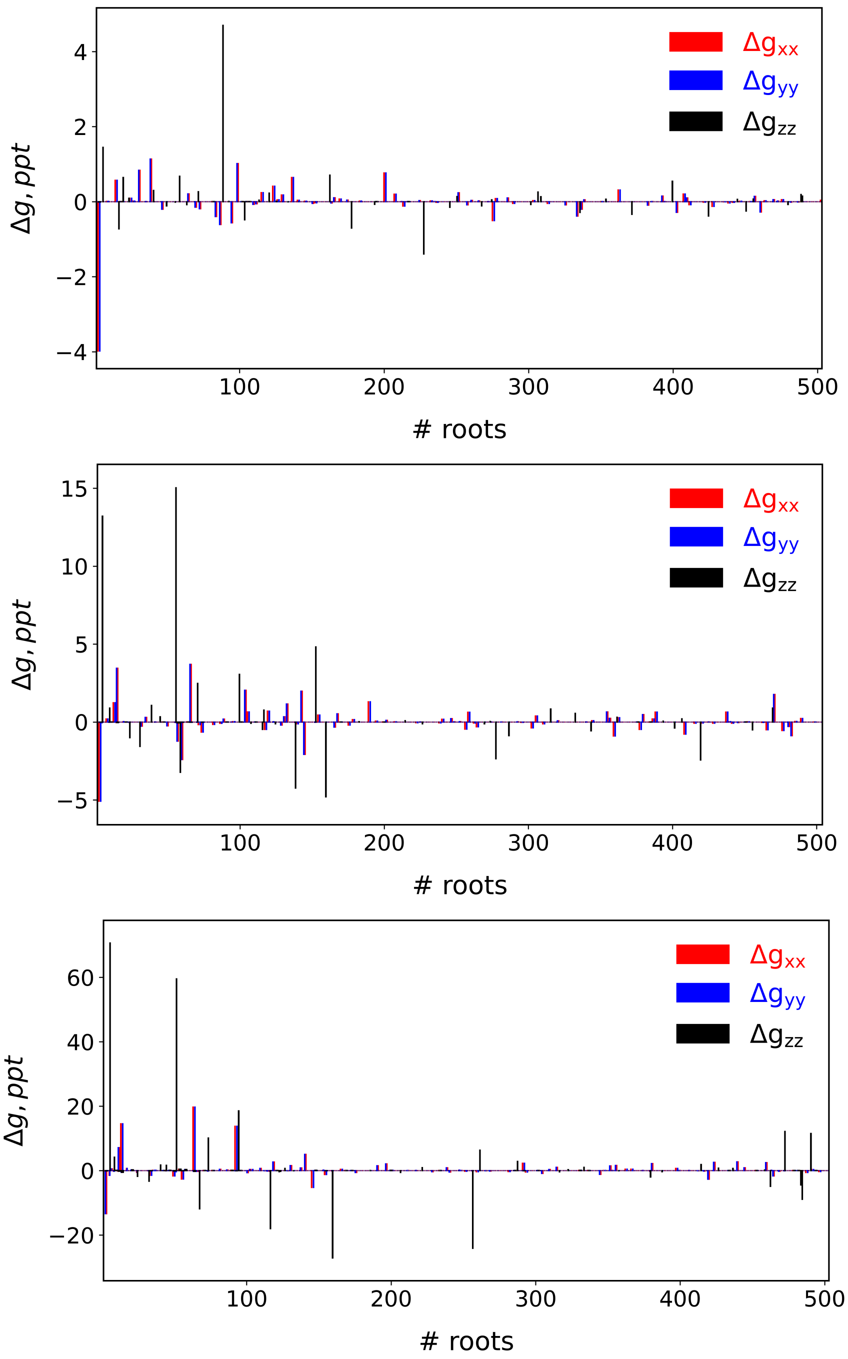

Figure S19: Δg components in two-state SOC-dressed Hamiltonians, the ground and a single excited state (# roots in the x-axis) for $MoOX_4^-$. From upper to lower, X=F,Cl,Br

6.2 Spin density plots

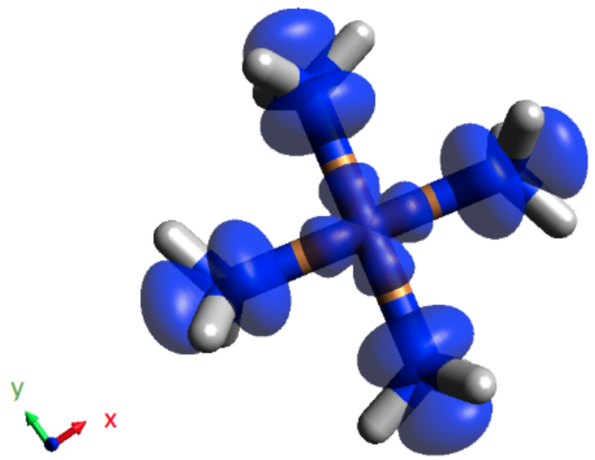

Figure S20: Spin density distribution (isovalue of 0.005 ea_0^{-3} of $[Cu(NH_3)_4]^{2+}$ spin doublet ground state computed at the B3LYP/def2-TZVP level.

6.3 Comparison of common gauge origin (CGO) and gauge including atomic orbitals (GIAOs)

Table S6: Comparison of CPKS Δg values in transition atom complexes using the center of nuclear charge as common gauge origin (CGO) or the gauge including atomic orbitals (GIAOs).

Molecule	Δg	CGO	GIAOs
$[CrOBr_4]^-$	$\Delta g_\perp$	-32.20	-31.48
	$\Delta g_\parallel$	120.52	121.11
$[CrOCl_4]^-$	$\Delta g_\perp$	-26.17	-26.02
	$\Delta g_\parallel$	17.71	17.74
$[CrOF_4]^-$	$\Delta g_\perp$	-31.12	-31.03
	$\Delta g_\parallel$	-22.35	-22.29
$[CuCl_4]^{2-}$	$\Delta g_\perp$	39.98	39.97
	$\Delta g_\parallel$	141.62	140.93
$[Cu(NH_3)_4]^{+2}$	$\Delta g_\perp$	40.27	40.13
	$\Delta g_\parallel$	146.82	146.42
$[MoOBr_4]^-$	$\Delta g_\perp$	4.91	5.56
	$\Delta g_\parallel$	128.59	128.57
$[MoOCl_4]^-$	$\Delta g_\perp$	-0.98	-0.84
	$\Delta g_\parallel$	28.94	28.91
$[MoOF_4]^-$	$\Delta g_\perp$	-2.77	-2.69
	$\Delta g_\parallel$	7.13	7.09
$[Ni(mnt)_2]^-$	$\Delta g_\perp$	62.54	–
	$\Delta g_\parallel$	103.37	–
TiF_3	$\Delta g_\perp$	-46.07	-46.04
	$\Delta g_\parallel$	-1.26	-1.25
$[VOBr_4]^{-2}$	$\Delta g_\perp$	-21.93	-21.58
	$\Delta g_\parallel$	56.54	57.12
$[VOCl_4]^{-2}$	$\Delta g_\perp$	-22.12	-22.02
	$\Delta g_\parallel$	-22.27	-21.68
$[VOF_4]^{-2}$	$\Delta g_\perp$	-27.87	-27.66
	$\Delta g_\parallel$	-41.35	-41.25

6.4 Exchange-correlation functional dependence

TDA results using B3LYP and BLYP functionals show marked differences: B3LYP yields values closer to experiment in some cases ($\Delta g_\perp$ in TiF_3, $\Delta g_\parallel$ in $[CuCl_4]^{2-}$, $\Delta g_\parallel$ in $[Cu(NH_3)_4]^{2+}$), while BLYP performs better in others ($\Delta g_\perp$ in $[VOF_4]^{2-}$, $\Delta g_\perp$ in $[CrOF_4]^{2-}$, Δg_{xx} in $[Ni(mnt)_2]^-$). Despite the good performance of B3LYP compared to other functionals, it is well known that

in transition metal complexes both functional dependence and system-dependence are more pronounced than in light atoms.

Table S7: Calculated Δg values (in ppt) for transition metal complexes molecules using in CPKS, TDA and full TDDFT, both with the B3LYP and BLYP functionals, using def2-TZVP basis set.

		B3LYP			BLYP		
molecule		CPKS	TDA	TDDFT	CPKS	TDA	Exp.[a]
TiF_3	$\Delta g_{\parallel}$	-1.3	15.8	15.7	-1.1	14.4	-11.1, -3.72
	$\Delta g_{\perp}$	-46.1	-57.8	-61.4	-32.8	-38.5	-111.3, -123.72
$[Ni(mnt)_2]^-$	Δg_{xx}	103.4	72.0	74.5	86.8	80.7	157.7
	Δg_{yy}	62.5	17.8	18.1	33.9	20.1	39.7
	Δg_{zz}	-5.5	-18.6	-18.6	-12.9	0.1	-4.3
$[CuCl_4]^{2-}$	$\Delta g_{\parallel}$	141.6	162.4	165.6	93.9	120.2	230.3
	$\Delta g_{\perp}$	40.0	23.8	23.9	30.6	25.8	46.7
$[Cu(NH_3)_4]^{2+}$	$\Delta g_{\parallel}$	146.8	166.9	169.9	100.8	121.1	238.7
	$\Delta g_{\perp}$	40.3	30.1	30.5	28.1	24.0	44.7
$[VOF_4]^{2-}$	$\Delta g_{\parallel}$	-41.4	-53.2	-54.5	-32.6	-51.5	-70.5
	$\Delta g_{\perp}$	-27.9	-18.6	-19.6	-24.0	-25.6	-30.5
$[VOCl_4]^{2-}$	$\Delta g_{\parallel}$	-22.3	-24.5	-25.7	-11.5	-19.3	-54.5
	$\Delta g_{\perp}$	-22.1	-15.7	-16.3	-17.9	-18.9	-23.3
$[VOBr_4]^{2-}$	$\Delta g_{\parallel}$	56.5	43.6	44.3	70.1	65.6	-
	$\Delta g_{\perp}$	-21.9	2.9	2.2	-16.7	-17.8	-
$[CrOF_4]^-$	$\Delta g_{\parallel}$	-22.4	-33.1	-33.7	-16.2	-24.1	-43.3
	$\Delta g_{\perp}$	-31.1	-10.1	-11.0	-26.0	-28.4	-34.3
$[CrOCl_4]^-$	$\Delta g_{\parallel}$	17.71	-3.8	-3.8	58.2	12.6	-13.3
	$\Delta g_{\perp}$	-26.2	-4.3	-4.8	67.02	-22.2	-26.3
$[CrOBr_4]^-$	$\Delta g_{\parallel}$	120.5	93.7	94.1	224.9	56.0	-
	$\Delta g_{\perp}$	-32.2	-8.2	-8.3	241.6	-55.5	-
$[MoOF_4]^-$	$\Delta g_{\parallel}$	7.1	5.5	5.5	7.4	2.1	-107.5
	$\Delta g_{\perp}$	-2.8	-0.5	-0.5	-2.5	-1.9	-77.0
$[MoOCl_4]^-$	$\Delta g_{\parallel}$	28.9	21.3	21.5	28.9	30.0	-37.3
	$\Delta g_{\perp}$	-1.0	5.1	5.1	-0.1	0.4	-56.1
$[MoOBr_4]^-$	$\Delta g_{\parallel}$	128.6	94.0	94.8	127.2	137.0	-
	$\Delta g_{\perp}$	4.9	50.3	50.1	8.9	29.7	-

[a]Experimental values from: ref. S6 $[CuCl_4]^{2-}$, ref. S7 $[Cu(NH_3)_4]^{2+}$, ref. S8 TiF_3, ref. S9 $[VOF_4]^{2-}$, $[MoOF_4]^-$, $[MoOCl_4]^-$, ref. S10 $[VOCl_4]^{2-}$, ref. S11 $[CrOF_4]^-$, ref. S12 $[CrOCl_4]^-$, ref. S13 $[Ni(mnt)_2]^-$.

7 Convergence analysis

Table S8: Convergence analysis of g-shift for TDDFT/TDA calculations with 100, 300, and 500 states. The last column shows the relative difference between 100 and 500 states.

Molecule	Δg (ppt)	100 states	300 states	500 states	Rel. Diff. (%)
$[CrOBr_4]^-$	$\Delta g_\perp$	-20.515	-2.453	-8.235	59.86
	$\Delta g_\parallel$	98.279	98.57	93.726	4.63
$[CrOCl_4]^-$	$\Delta g_\perp$	-12.872	-4.355	-4.252	66.97
	$\Delta g_\parallel$	11.45	-0.736	-3.793	133.13
$[CrOF_4]^-$	$\Delta g_\perp$	-17.44	-8.897	-10.125	41.94
	$\Delta g_\parallel$	-28.474	-31.792	-33.138	-16.38
$[CuCl_4]^{2-}$	$\Delta g_\perp$	28.064	24.65	23.767	15.31
	$\Delta g_\parallel$	176.481	166.755	162.443	7.95
$[Cu(NH_3)_4]^{+2}$	$\Delta g_\perp$	31.134	30.507	30.101	3.32
	$\Delta g_\parallel$	172.782	168.899	166.873	3.42
$[MoOBr_4]^-$	$\Delta g_\perp$	32.025	40.998	50.254	-56.92
	$\Delta g_\parallel$	145.407	86.57	93.986	35.36
$[MoOCl_4]^-$	$\Delta g_\perp$	-1.583	4.578	5.122	423.56
	$\Delta g_\parallel$	29.901	22.364	21.322	28.69
$[MoOF_4]^-$	$\Delta g_\perp$	-2.241	0.067	-0.448	80.01
	$\Delta g_\parallel$	7.159	5.319	5.448	23.90
$[Ni(mnt)_2]^-$	Δg_{xx}	70.3	–	72.0	2.42
	Δg_{yy}	22.1	–	17.8	-19.46
	Δg_{zz}	-5.6	–	-18.6	232.14
TiF_3	$\Delta g_\perp$	-57.474	-57.812	-57.776	-0.53
	$\Delta g_\parallel$	-57.428	-57.764	-57.705	-0.48
$[VOBr_4]^{-2}$	$\Delta g_\perp$	-24.249	-2.84	2.864	111.81
	$\Delta g_\parallel$	38.519	50.867	43.589	-13.16
$[VOCl_4]^{-2}$	$\Delta g_\perp$	-24.768	-16.772	-15.688	36.66
	$\Delta g_\parallel$	-32.823	-26.707	-24.439	25.54
$[VOF_4]^{-2}$	$\Delta g_\perp$	-20.016	-17.935	-18.632	6.91
	$\Delta g_\parallel$	-60.281	-52.487	-53.195	11.75
NBr	$\Delta g_\perp$	12.038	12.325	11.294	-6.18
	$\Delta g_\parallel$	-2.302	-2.583	-2.611	-11.83
SeO	$\Delta g_\perp$	9.362	-	9.618	-2.73
	$\Delta g_\parallel$	-1.042	-	-1.162	-11.5

8 g-shifts with BLYP functional

Table S9: Comparison of Δg values (in ppt) for CPKS and TDA methods using BLYP functional.

Molecule	Δg (ppt)	CPKS	TDA
CdH	$\Delta g_{\perp}$	-91.79	-96.759
	$\Delta g_{\parallel}$	0.14	-3.058
$CO_2{}^-$	$\Delta g_{\perp}$	-0.61	0.631
	$\Delta g_{\parallel}$	-4.93	-0.377
$CrOBr_4{}^-$	$\Delta g_{\perp}$	241.63	-55.474
	$\Delta g_{\parallel}$	241.63	56.041
$CrOCl_4{}^-$	$\Delta g_{\perp}$	67.02	-21.513
	$\Delta g_{\parallel}$	67.02	12.579
$CrOF_4{}^-$	$\Delta g_{\perp}$	-25.95	-28.418
	$\Delta g_{\parallel}$	-25.95	-24.099
$CuCl_4{}^{-2}$	$\Delta g_{\perp}$	30.56	25.773
	$\Delta g_{\parallel}$	30.56	120.228
$CuNH_3$	$\Delta g_{\perp}$	28.12	23.974
	$\Delta g_{\parallel}$	28.13	121.097
H_2O^+	$\Delta g_{\perp}$	4.10	-0.005
	$\Delta g_{\parallel}$	11.50	4.253
HgH	$\Delta g_{\perp}$	273.82	–
	$\Delta g_{\parallel}$	12313.71	–
IrH_2	$\Delta g_{\perp}$	-321.78	–
	$\Delta g_{\parallel}$	3.44	–
$MoOBr_4{}^-$	$\Delta g_{\perp}$	8.85	29.743
	$\Delta g_{\parallel}$	8.85	137.031
$moocl_4{}^-$	$\Delta g_{\perp}$	-0.14	0.401
	$\Delta g_{\parallel}$	-0.14	30.007
$moof_4{}^-$	$\Delta g_{\perp}$	-2.45	-1.906

Molecule	Δg (ppt)	CPKS	TDA
	$\Delta g_{\|\|}$	-2.45	2.095
NBr	$\Delta g_{\perp}$	22.49	8.963
	$\Delta g_{\|\|}$	-0.06	0.181
NI	$\Delta g_{\perp}$	56.08	12.774
	$\Delta g_{\|\|}$	0.03	-23.491
$nimnt_2{}^-$	$\Delta g_{\perp}$	-12.93	80.654
	$\Delta g_{\|\|}$	33.88	0.131
NO_2	$\Delta g_{\perp}$	-0.58	2.722
	$\Delta g_{\|\|}$	-10.48	-0.712
PdH	$\Delta g_{\perp}$	135.12	–
	$\Delta g_{\|\|}$	0.06	–
RhH_2	$\Delta g_{\perp}$	491.08	-99.592
	$\Delta g_{\|\|}$	485.15	433.755
TiF_3	$\Delta g_{\perp}$	-32.75	-38.5
	$\Delta g_{\|\|}$	-1.1	14.356
vobr−2	$\Delta g_{\perp}$	-16.67	-17.813
	$\Delta g_{\|\|}$	-16.67	65.546
$vocl_4{-}2$	$\Delta g_{\perp}$	-17.88	-18.888
	$\Delta g_{\|\|}$	-17.88	-19.27
$vof_4{-}2$	$\Delta g_{\perp}$	-23.96	-25.578
	$\Delta g_{\|\|}$	-23.96	-51.519

References

(S1) Carreras, A. PyQchem: a python interface for Q-Chem. `https://github.com/abelcarreras/PyQchem`, 2019.

(S2) Tatchen, J.; Kleinschmidt, M.; Marian, C. M. Calculating electron paramagnetic res-

onance g-matrices for triplet state molecules from multireference spin-orbit configuration interaction wave functions. *J. Chem. Phys.* **2009**, *130*, 154106.

(S3) Neese, F. Software update: The ORCA program system—Version 5.0. *WIREs: Comput. Mol. Sci.* **2022**, *12*, 414.

(S4) Najibi, A.; Goerigk, L. The nonlocal kernel in van der Waals density functionals as an additive correction: An extensive analysis with special emphasis on the B97M-V and ωB97M-V approaches. *J. Chem. Theory Comput.* **2018**, *14*, 5725–5738.

(S5) Cebreiro-Gallardo, A.; Casanova, D. *Phys. Chem. Chem. Phys.*, 2025, DOI: 10.1039/D4CP04511D.

(S6) Chow, C.; Chang, K.; Willett, R. Electron spin resonance spectra and covalent bonding in the square-planar CuCl 4 2- and CuBr 4 2- ions. *J. Chem. Phys.* **1973**, *59*, 2629–2640.

(S7) Scholl, H.; Hüttermann, J. ESR and ENDOR of copper (II) complexes with nitrogen donors: probing parameters for prosthetic group modeling of superoxide dismutase. *J. Phys. Chem.* **1992**, *96*, 9684–9691.

(S8) De Vore, T.; Weltner Jr, W. Titanium difluoride and titanium trifluoride molecules: electron spin resonance spectra in rare-gas matrices at 4 K. *J. Am. Chem. Soc.* **1977**, *99*, 4700–4703.

(S9) Sunil, K.; Rogers, M. ESR studies of some oxotetrahalo complexes of vanadium (IV) and molybdenum (V). *Inorg. Chem.* **1981**, *20*, 3283–3287.

(S10) Flowers, J.; Hempel, J.; Hatfield, W.; Dearman, H. An EPR study of VO2+ and Cr3+ in (NH4) 2SbCl5. *J. Chem. Phys.* **1973**, *58*, 1479–1486.

(S11) Manoharan, P.; Rogers, M. T. Ligand hyperfine interactions in molybdenyl and chromyl halide complexes. *J. Chem. Phys.* **1968**, *49*, 5510–5519.

(S12) Amorelli, A.; Amorelli, T. S.; Evans, J. C.; Rowlands, C. C. An EPR study of the interaction of sulphur dioxide, chlorine and hydrogen chloride with transition metal impregnated rutile TiO 2. *J. Mater. Sci.* **1990**, *25*, 796–800.

(S13) Maki, A.; Edelstein, N.; Davison, A.; Holm, R. Electron Paramagnetic Resonance Studies of the Electronic Structures of Bis (maleonitriledithiolato) copper (II),-nickel (III),-cobalt (II), and-rhodium (II) Complexes. *J. Am. Chem. Soc.* **1964**, *86*, 4580–4587.
